# Mapping the Evolutionary Landscape of Solid Tumor Immunotherapy: A Quarter-Century Bibliometric Analysis of the Title-Defined Core Literature (2000-2025)

**DOI:** 10.7150/ijms.137251

**Published:** 2026-07-22

**Authors:** Ender Eren OZCELIK, Gul AKIN, Hulya ODABASI BUKUN, Mursel SALI, Ahmet Bilgehan SAHIN, Adem DELIGONUL, Erdem CUBUKCU, Turkkan EVRENSEL

**Affiliations:** Department of Medical Oncology, School of Medicine, Bursa Uludag University, Bursa 16059, Turkey.

**Keywords:** cancer immunotherapy, bibliometric analysis, solid tumors, tumor microenvironment, neoadjuvant therapy, checkpoint inhibitors

## Abstract

Cancer immunotherapy has evolved from an experimental niche to the established fourth pillar of oncological care. While the clinical success of checkpoint inhibitors is well-documented, the macro-level evolutionary trajectory of the field remains unmapped. This study provides a quarter-century bibliometric analysis of the core title-defined research landscape in solid tumor immunotherapy. Data from the Web of Science Core Collection were utilized as the primary source via a title-restricted search strategy, and core thematic structures were cross-compared against a high-impact Scopus subset. While this strategy ensures high clinical specificity, it represents a primary methodological limitation by inherently reducing search sensitivity and potentially excluding relevant studies with non-descriptive titles. The final data retrieval was conducted on January 1, 2026. The study covered the period from January 1, 2000, to December 31, 2025, utilizing advanced network visualization techniques via VOSviewer and Biblioshiny to identify spatiotemporal trends. 54,546 documents were identified, revealing a rapid and sustained expansion in publication volume, a descriptive trend that chronologically aligns with major clinical milestones such as the 2011 FDA approval of ipilimumab. Geopolitically, a distinctive divergence emerged: while China has surpassed the USA in total publication volume, the USA maintains higher integration in global collaboration networks and greater per-article citation counts. Network analysis visualized a decisive conceptual evolution from the "Cytokine Era" (2000-2010) to the "Checkpoint Revolution" (2011-2019). Most critically, recent trends (2020-2025) indicate a clear shift in research toward the "Tumor Microenvironment" and "Neoadjuvant Therapy" with the latter emerging as a predominant focus of recent translational research. The field's research focus is currently undergoing a structural transition from monotherapy in metastatic settings to combinatorial and perioperative strategies in early-stage disease. Current bibliometric trends indicate a growing academic focus on overcoming resistance mechanisms in "cold" tumors, reflecting strong translational interest in combining and sequencing immunotherapies with next-generation modalities, such as antibody-drug conjugates.

## 1. INTRODUCTION

The landscape of modern oncology has undergone significant changes over the past quarter-century 1. While surgery, chemotherapy, and radiotherapy traditionally constituted the "three pillars" of cancer treatment, the advent of cancer immunotherapy has firmly established itself as the fourth pillar, marking a paradigm shift in the management of solid tumors 2,3. Unlike direct cytotoxic agents, immunotherapeutic modalities leverage the host's immune system to recognize and eliminate malignant cells, a concept rooted in the foundational "Cancer Immunity Cycle" described by Chen and Mellman 4. The translation of this biological understanding into clinical practice — most notably through the discovery of immune checkpoint inhibitors (ICIs) targeting CTLA-4 and the PD-1/PD-L1 axis — has led to unprecedented durable responses in historically refractory malignancies, such as metastatic melanoma and non-small cell lung cancer (NSCLC) 5,6.

The period from 2000 to 2025 represents a critical epoch in this evolution. The early 2000s were characterized by the limited efficacy of cytokine-based therapies and initial skepticism about cancer vaccines. However, the landmark FDA approval of ipilimumab in 2011, followed by nivolumab and pembrolizumab, chronologically paralleled a marked and sustained acceleration in research activity 7. This momentum was further recognized by the 2018 Nobel Prize in Physiology or Medicine awarded to James P. Allison and Tasuku Honjo, validating the therapeutic potential of negative immune regulation 8.

As we approach the end of this quarter-century, the research landscape is evolving from a "monotherapy-centric" model to an investigational focus on precision combinations. While adoptive cell therapies, such as CAR-T, have revolutionized hematology, their success in solid tumors remains limited by the hostile tumor microenvironment (TME) 9. Consequently, recent publication trends have shifted towards investigating synergistic strategies to overcome resistance. This includes exploring the integration of ICIs with novel modalities, such as bispecific antibodies (BiTEs) and, increasingly, antibody-drug conjugates (ADCs). Although ADCs are distinct from classical immunotherapy, their emerging role as "targeted delivery vectors" that can induce immunogenic cell death (ICD) and potentiate immune responses has become a major focus of translational research in solid tumors. 10,11.

This rapid clinical and translational success has coincided with a substantial and sustained expansion in scientific literature. The sheer volume of publications, ranging from preclinical mechanistic studies to large-scale Phase III trials, has created an "information bottleneck", making it increasingly challenging for clinicians and researchers to synthesize the global landscape effectively. Traditional systematic reviews or meta-analyses, while valuable, often focus on specific clinical questions and fail to capture the macroevolutionary trajectory of the field as a whole 12.

In this context, bibliometrics emerges as a powerful methodological approach to navigate this vast ocean of data. By applying quantitative statistical analysis to bibliographic records, bibliometrics enables mapping conceptual structures, identifying research hotspots, and visualizing collaboration networks across countries and institutions 13. While previous bibliometric analyses have evaluated segments of the immuno-oncology landscape, they are predominantly restricted to distinct hematological malignancies (e.g., diffuse large B-cell lymphoma), limited to single solid tumor types (e.g., esophageal cancer), confined to specific treatment modalities such as cancer vaccines, or strictly focused on isolated biological phenomena like tumor immune escape 14-17. These narrower frameworks are inherently unable to capture the critical "pan-tumor cross-pollination" of immunological concepts across diverse oncological disciplines. Furthermore, the structural novelty of the present study extends beyond its extensive quarter-century scale; it uniquely maps the macro-level topological transition of the title-defined core literature in the solid-tumor field from historical palliative monotherapies to the highly complex combinatorial and neoadjuvant strategies currently dominating the tumor microenvironment (TME) research space. To explicitly delineate our conceptual novelty, Table 1 summarizes these pivotal prior bibliometric approaches and compares them with the structural and methodological scope of the present study.

This study aims to map the title-defined core literature of cancer immunotherapy research in solid tumors over a definitive quarter-century period (January 1, 2000 - December 31, 2025). Utilizing the Web of Science Core Collection (WoSCC), we seek to: (i) quantify the global scientific production and growth trends; (ii) identify the most influential countries, institutions, and journals driving this revolution; (iii) visualize the evolution of research themes from the "cytokine era" to the "checkpoint era" and the emerging "combinatorial era"; and (iv) synthesize current bibliometric visibility to offer hypothesis-generating interpretations regarding emerging research momentum.

## 2. METHODS

### 2.1. Data Source and Index Selection

This bibliometric analysis was conducted using data retrieved from the Web of Science Core Collection (WoSCC). To ensure the dataset focused strictly on high-quality clinical and biological research while capturing emerging trends, the search was restricted to the Science Citation Index Expanded (SCI-EXPANDED) and Emerging Sources Citation Index (ESCI). The Social Sciences Citation Index (SSCI) and Arts & Humanities Citation Index (AHCI) were excluded to maintain a homogeneous focus on medical and life sciences.

**Figure 1:**
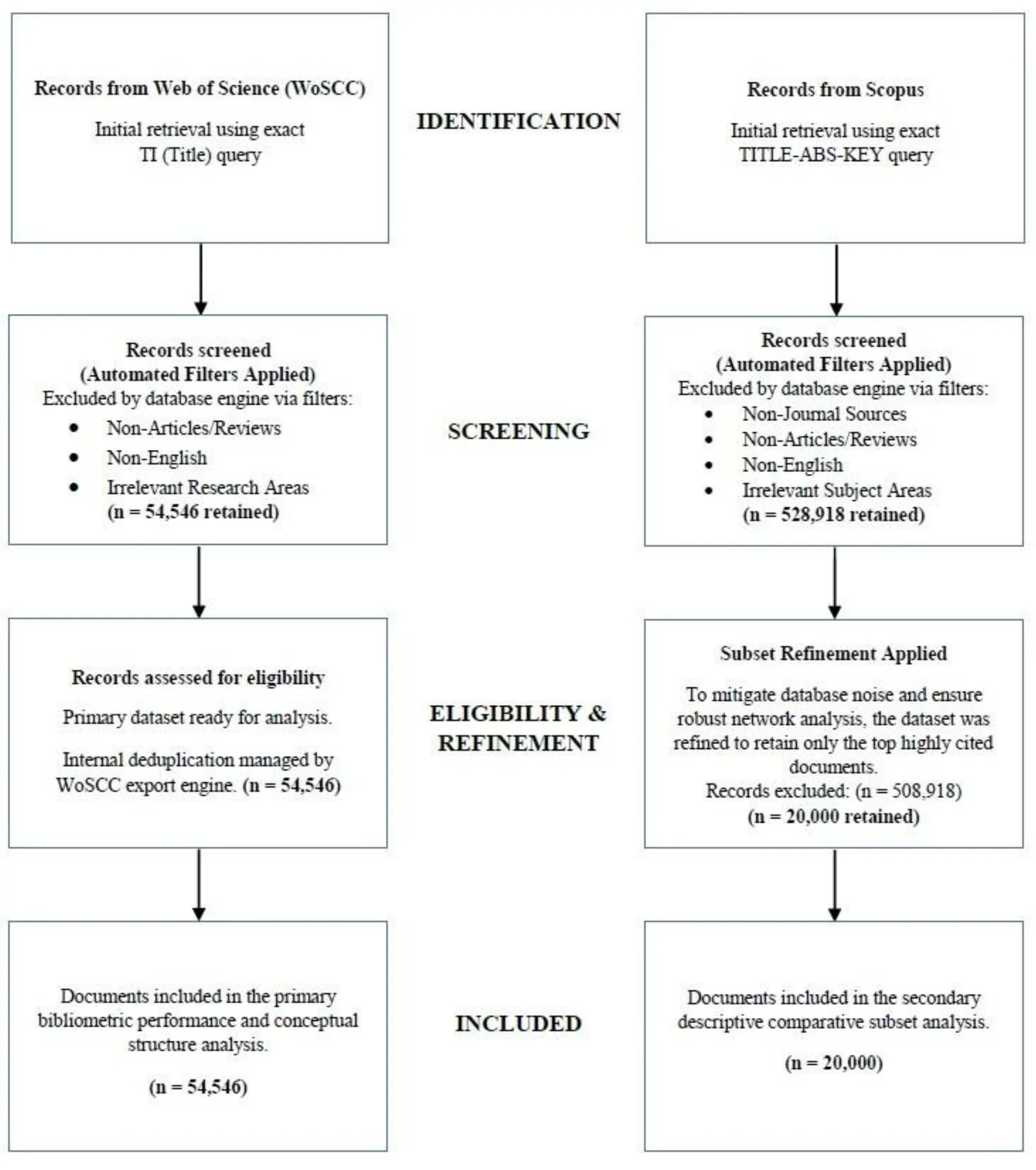
** Database retrieval and dataset construction flowchart**.

The entire data retrieval process, including dataset identification, automated screening, and inclusion, was systematically executed and documented. The precise steps of dataset construction are detailed in the retrieval flowchart (Figure 1). The retrieval was performed on a single day (January 1, 2026) to prevent discrepancies arising from daily database updates, covering the quarter-century period from January 1, 2000, to December 31, 2025.

### 2.2. Search Strategy and "Solid Tumor" Focus

A rigorous, multi-step search strategy was designed to identify original research and reviews specifically defining the landscape of immuno-oncology in solid tumors. To maximize specificity and eliminate false-positive results (e.g., incidental mentions in abstracts), the search query was restricted to the "Title" (TI) field. The complete search strings, Boolean operators, and detailed syntax for each dataset are provided in Supplementary Table S1.

The search strategy consisted of three strategic layers:

**Immunotherapy Agents (Inclusive & Global):** This set included general terms (e.g., "checkpoint inhibitor", "CAR-T"), standard FDA/EMA-approved agents (e.g., pembrolizumab, nivolumab), and novel modalities (e.g., bispecifics, TCR-T, TIL therapy). To ensure a truly global perspective, the search strategy incorporated a comprehensive list of immuno-oncology agents approved by major international regulatory authorities, including but not limited to the FDA (USA), EMA (Europe), NMPA (China), and PMDA (Japan). This approach ensured the capture of pivotal studies from diverse geographic regions, regardless of the agent's regulatory origin. Standardized generic drug names were used for all specific agents.

**Solid Tumor Identification:** This set included general oncology terms and specific solid malignancies (e.g., "glioblastoma", "melanoma", "mesothelioma") to capture studies that might not use the generic "cancer" suffix.

**Exclusion of Hematological Malignancies:** To isolate the solid tumor landscape, a "NOT" operator was employed to rigorously exclude publications with titles containing leukemia, lymphoma, myeloma, or general hematologic terms.

To rigorously justify the exclusive use of the Title (TI) field, we conducted a manual sensitivity analysis to quantify the recall-versus-specificity trade-off. We compared our finalized search string using the 'Title' (TI) field against the broader 'Topic' (TS) field. Using simple random sampling across the entire 2000-2025 study period, we randomly selected 100 records captured by the broader TS search but excluded by our strict TI strategy. Each record was independently assessed by two clinician co-authors and classified as a false positive if the study's primary focus was not the direct clinical investigation or mechanistic study of immunotherapy in solid tumors. This structured review revealed an 84% non-specific record rate within the TS-only records. Specifically, the vast majority were irrelevant to the field's core structural mapping, comprising studies in which immunotherapy agents were mentioned only incidentally in the abstract background, cross-disciplinary engineering reports, or hematological studies that referenced solid tumors in passing. While the broader TS strategy increased total retrieval, introducing a noise rate of over 80% in a large-scale analysis of >54,000 documents would have compromised the topological integrity of VOSviewer clustering (e.g., by blurring the boundaries of distinct clusters such as 'Tumor Microenvironment' or 'Neoadjuvant'). Consequently, the TI-only strategy was selected to maximize clinical specificity by mapping the field's dedicated, highly focused core literature.

### 2.3. Data Refinement and Inclusion Criteria

Following the initial retrieval, the dataset underwent strict refinement to ensure clinical relevance.

**Document Types:** Only "Articles" (original research) and "Reviews" were included. Editorial materials, meeting abstracts, letters, and corrections were excluded from the analysis.

**Language:** Restricted to English.

**Research Areas Filter:** To remove irrelevant records (e.g., engineering, veterinary sciences, psychology), the dataset was filtered to include only clinically relevant WoS categories: Oncology, Immunology, Pharmacology Pharmacy, Cell Biology, Medicine Research Experimental, Biochemistry Molecular Biology, and Medicine General Internal.

After applying these filters, a total of 54546 records were identified. The full bibliographic records, including titles, authors, abstracts, keywords, and cited references, were exported in "Plain Text" format for downstream analysis.

Since the primary dataset was exported exclusively from WoSCC via a single continuous query, internal database deduplication was handled by the Web of Science export engine. No manual deduplication was required for the primary WoSCC analysis. The final refined dataset yielded 54,546 records. Furthermore, because the final data retrieval was conducted on January 1, 2026, it is recognized that the indexing of publications from the final quarter of 2025 may be incomplete across the databases. Therefore, the absolute publication counts for the year 2025 should be interpreted as near-final estimates subject to minor indexing delays.

### 2.4. Bibliometric Analysis and Visualization

Data processing and statistical analysis were performed using the R programming language (Version 4.5.2) and VOSviewer (Version 1.6.20) 18.

The primary descriptive bibliometric analysis was conducted using the "bibliometrix" R package (version 5.2.1) and its web-based interface, Biblioshiny^19^. This tool was utilized to calculate:

**Performance Analysis:** Annual scientific production, growth rates, and the most productive countries, institutions, and authors.

**Source Impact:** Application of Bradford's Law to identify core journals in the field.

**Conceptual Structure:** Generation of Trend Topics and Thematic Maps to visualize the evolution of research themes.

Additionally, VOSviewer was employed to construct and visualize complex bibliometric networks. To ensure full methodological reproducibility, the exact computational settings and harmonization protocols were standardized as follows:

**Keyword Co-occurrence Analysis:** The analysis prioritized "Author Keywords" (DE) over "Keywords Plus" (ID) to capture the authors' exact conceptual intent and minimize algorithmic indexing noise. A "full counting" method was applied. To optimize network visualization and isolate core thematic pillars, a minimum occurrence threshold of 250 was established for the WoSCC dataset. Network normalization was performed using the default "Association strength" method, with the clustering resolution parameter set to 1.00.

**Thesaurus and Synonym Harmonization:** Before network generation, extensive data cleaning was performed using a manually curated VOSviewer thesaurus file (thesaurus.txt). This step was critical to merge singular/plural forms, standardize complex acronyms (e.g., unifying "non-small cell lung cancer" and "NSCLC"), consolidate specific drug targets (e.g., unifying "PD-L1", "programmed death-ligand 1"), and eliminate generic stopwords (e.g., "patients", "survival", "in vitro") that lack specific thematic value. The complete thesaurus file detailing all synonym merging is provided in the Supplementary material.

This thesaurus-based harmonization was applied specifically to the construction of the VOSviewer keyword co-occurrence network (Figures 7 and 8). The Biblioshiny-generated descriptive visualizations (the three-field plot in Figure 5 and the trend topic analysis in Figure 6) were derived directly from the raw, un-harmonized 'Author Keywords' (DE) field; consequently, minor lexical variants (e.g., singular/plural forms such as 'immune checkpoint inhibitor' vs. 'immune checkpoint inhibitors') may appear as separate nodes in these specific figures, as Biblioshiny's three-field plot module does not currently support custom thesaurus integration. To prioritize macro-topological paradigm mapping over micro-biological resolution, class-level aggregations were applied: specific checkpoint targets (anti-PD-1, anti-PD-L1) were unified under 'immune checkpoint inhibitors', and T-cell subsets were consolidated under their parent term.

**Geographical and Institutional Metrics:** For the performance analysis of countries and institutions generated via the Biblioshiny interface (bibliometrix R package version 5.2.1 operating on R version 4.5.2), a "full counting" method was utilized. This approach ensures that every collaborating country or institution receives a full credit count for a multiauthor publication, accurately reflecting their absolute participation in global research networks. Before ranking, raw institutional affiliation strings were manually harmonized to prevent the artificial fragmentation of publication counts across overlapping organizational entities. Specifically, hospitals, cancer centers, and medical schools that operate as formally affiliated or constituent units of a parent university or university system (e.g., MD Anderson Cancer Center as a component of the University of Texas System; Harvard-affiliated teaching hospitals and faculties as components of Harvard University) were consolidated under a single parent institutional label, guided by each institution's publicly listed governance structure. This harmonization was applied consistently across the full 54,546-document dataset before generating Figure 4.

**Overlay Visualization Analysis:** To map the temporal evolution of the research landscape, network nodes were colored based on their average publication year.

Annual scientific production and the compound annual growth rate (CAGR) were calculated using the Biblioshiny interface. Independently, an Ordinary Least Squares (OLS) regression analysis was conducted to estimate the linear trend of publication growth over time. To rigorously characterize the temporal growth trajectory, annual publication counts were subjected to three complementary regression analyses. First, an overall log-linear exponential model was fitted across the entire 2000-2025 period using OLS on log-transformed counts, with R², 95% confidence intervals for the slope, and residuals reported. Second, a prespecified segmented regression was applied across the three clinically defined phases (Latent: 2000-2010; Awakening: 2011-2015; Acceleration: 2016-2025), with an independent log-linear OLS model fitted to each segment. Third, an exploratory change-point analysis was performed via exhaustive two-breakpoint grid search to identify the breakpoints that minimized the combined log-scale residual sum of squares across all three segments.

Following computational clustering, the final interpretation of the bibliometric data and cluster semantics was conducted by a senior medical oncologist. This expert annotation ensured clinical relevance and accurate thematic categorization without altering the algorithmic structure of the generated nodes or their topological labels.

### 2.5. Comparative Subset Analysis: Multi-Database Approach

To descriptively compare the thematic structure of foundational research themes identified in the primary Web of Science Core Collection (WoSCC) dataset, a comparative subset analysis was conducted using the Scopus database. Scopus was selected due to its substantially broader coverage of biomedical and pharmacological journals 20. Using an adapted database-specific search query (Supplementary Table S1), a total of 528,918 documents were identified. This order-of-magnitude discrepancy compared to the WoSCC dataset reflects the fundamental differences in database coverage and indexing depth. To mitigate extreme database noise and computationally enable high-quality network mapping 18, the Scopus dataset was refined to an analytical subset of the top 20,000 most-cited documents.

Since there is no direct field-equivalent to the WoSCC Title (TI) field in Scopus, the TITLE-ABS-KEY field — which simultaneously searches the title, abstract, and author keywords — was deliberately selected to identify the broadest possible pool of foundational literature for the comparative subset analysis. This field choice, rather than database coverage alone, primarily accounts for the order-of-magnitude difference in initial retrieval volume (528,918 vs. 54,546 documents). The exact, auditable Boolean search strings and specific filters applied for both WoSCC and Scopus are provided in Supplementary Table S1 to guarantee full methodological reproducibility 12. The search was executed within the publication timeframe of 2000 to 2025. To align the dataset with high-quality, finalized scientific output, the following inclusion criteria were strictly applied:

**Document Types:** Limited to "Article" and "Review" only.

**Language:** Restricted to "English."

**Publication Stage:** Limited to "Final" to exclude "Article in Press" records.

**Source Type:** Restricted to "Journal" sources.

**Subject Areas:** Filtered to include only relevant domains: “Medicine”; “Biochemistry, Genetics and Molecular Biology”; “Immunology and Microbiology”; “Pharmacology, Toxicology and Pharmaceutics”.

The retrieved metadata from Scopus were exported in CSV format and analyzed using VOSviewer (version 1.6.20) and Microsoft Excel to descriptively compare the major historical thematic structures and country-level productivity rankings observed in the primary WoSCC dataset.

To quantify the inter-database agreement, we computed a Spearman's rank correlation coefficient (ρ) to compare the productivity rankings of the top 10 contributing countries identified in the primary WoSCC dataset and the comparative Scopus subset. The analysis yielded a highly significant correlation (ρ = 0.90, p < 0.001), suggesting a high rank-order consistency in country-level productivity between the two databases. This statistical descriptive comparison underscores that our findings regarding the geopolitical research landscape are not artifacts of database selection but reflect stable, globally observable scientific trends. But this correlation should be interpreted with caution, as both datasets share known confounders.

### 2.6. Use of Generative AI and AI-Assisted Technologies

During manuscript preparation, the authors used Gemini 3 Flash solely to assist with English-language editing and grammar refinement of the author-drafted text to improve readability and clarity. The tool was applied only to the manuscript text. It was not used for study conceptualization, the design or execution of the search strategy, data retrieval or collection, bibliometric computation or statistical analysis, the interpretation of results, the formulation of conclusions, or the generation of any figures or images.

## 3. RESULTS

### 3.1. Global Scientific Production and Growth Trends

The bibliometric analysis of the title-defined core literature in the solid tumor immunotherapy landscape over the quarter-century period (2000-2025) identified a total of 54,546 documents published across 1,563 distinct academic sources. This substantial volume of literature reflects the transformative impact of immuno-oncology on clinical practice. The overall log-linear exponential model confirmed a robust growth trajectory across the full 2000-2025 period (annual growth rate: 13.4%, R²=0.876, p=2.3×10⁻¹², 95% CI for slope: 10.6%-15.3%), indicating sustained, accelerating scientific interest developing in parallel with the progressive approval of immune checkpoint inhibitors and novel combination strategies.

The analysis of collaboration patterns reveals that 20.87% of the documents involved international co-authorships, highlighting the global nature of this research domain. However, a significant portion of the output remains driven by domestic efforts within major powerhouse nations. The detailed descriptive characteristics of the dataset are summarized in Table 2.

**Figure 2:**
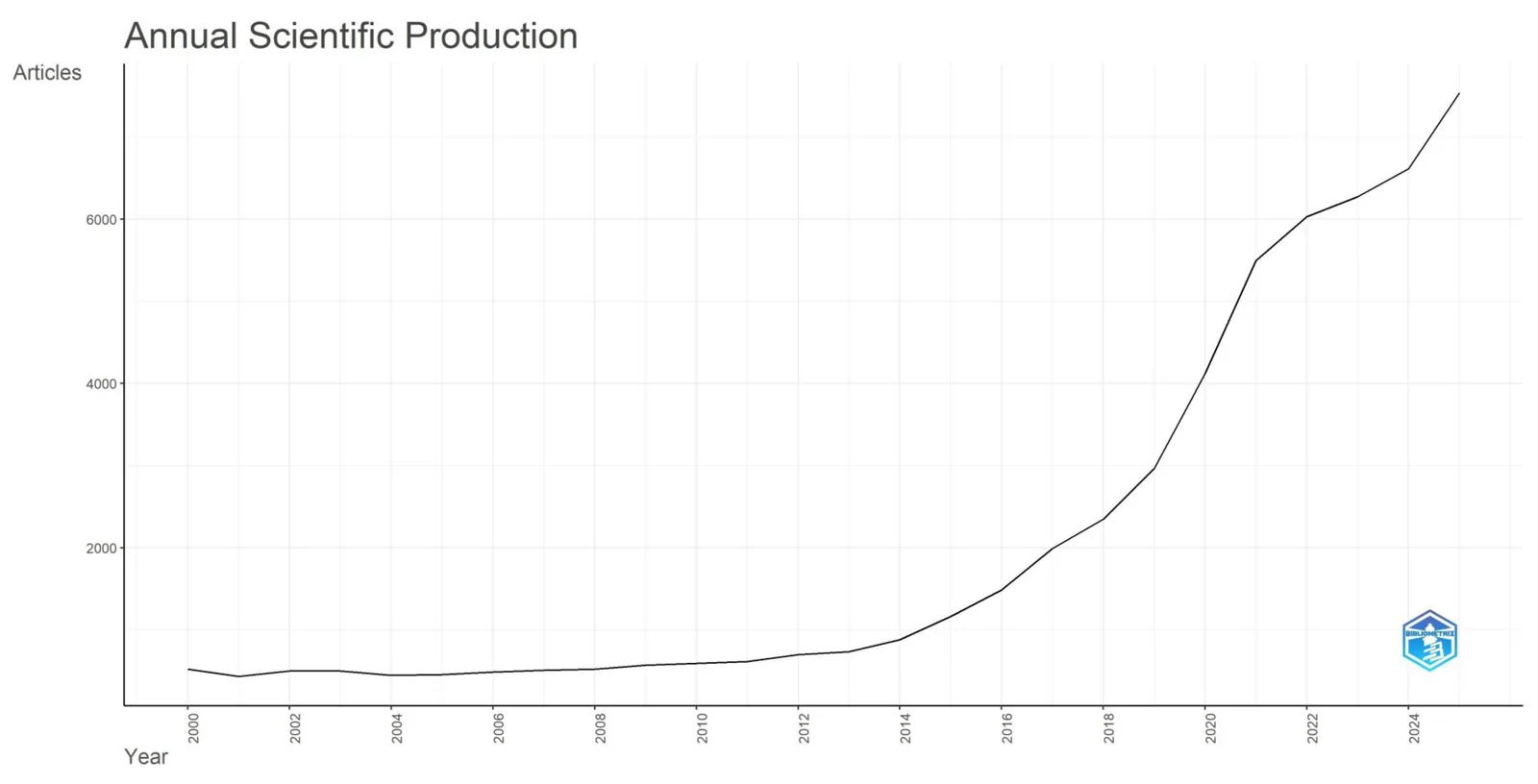
** Annual scientific production of the title-defined core literature in solid tumor immunotherapy (2000-2025).** The curve exhibits a sustained growth pattern, particularly following the widespread adoption of immune checkpoint inhibitors after 2015. (Note: The publication count for the terminal year 2025 represents a near-final estimate due to database indexing lags inherent to the data extraction date.)

Figure 2 illustrates annual scientific production, revealing a distinctive growth trajectory that mirrors the field's clinical milestones. The evolution of publication volume can be categorized into three phases:

**The Latent Phase (2000-2010):** A period of stagnation where annual output remained below 600 publications, coinciding with the limited clinical success of early cytokine-based therapies and cancer vaccines (+1.8%/yr, R²=0.39, p=0.041).

**The Awakening Phase (2011-2015):** A gradual increase was observed following the FDA approval of ipilimumab (2011) and the initial pivotal trials of PD-1 inhibitors, signaling a renewed optimism in the field (+16.3%/yr, R²=0.928, p=0.008).

**The Acceleration Phase (2016-2025):** A sharp and continuous surge in publication output occurred, a trajectory that chronologically parallels the broad regulatory approvals of nivolumab and pembrolizumab across multiple indications, as well as the awarding of the 2018 Nobel Prize in Physiology or Medicine. The descriptive trend shows no signs of plateauing, underscoring the continued expansion of the immuno-oncology landscape into combinatorial and neoadjuvant settings. (+20.2%/yr, R²=0.942, p<0.001). Notably, a complementary data-driven change-point analysis identified optimal breakpoints at 2013 and 2021, suggesting that the publication response to the 2011 ipilimumab approval exhibited a 1-2 year indexing and dissemination lag. The 2013-2020 interval represented the most concentrated growth phase (R²=0.997, +27.7%/yr), followed by an apparent relative deceleration across the 2021-2025 segment (+7.5%/yr). This terminal slope should be interpreted with particular caution: because the final data extraction was performed on January 1, 2026, the 2025 index year was still incomplete at the time of retrieval (a near-final estimate), and this terminal-year undercount is expected to bias the 2021-2025 growth estimate downward. The post-2021 figure should therefore not be read as evidence of a true plateau or decline in research output; a degree of thematic consolidation within the most prevalent checkpoint-blockade categories may additionally contribute, but the present data cannot separate these two effects.

### 3.2. Geographical Distribution and International Collaboration

**Figure 3:**
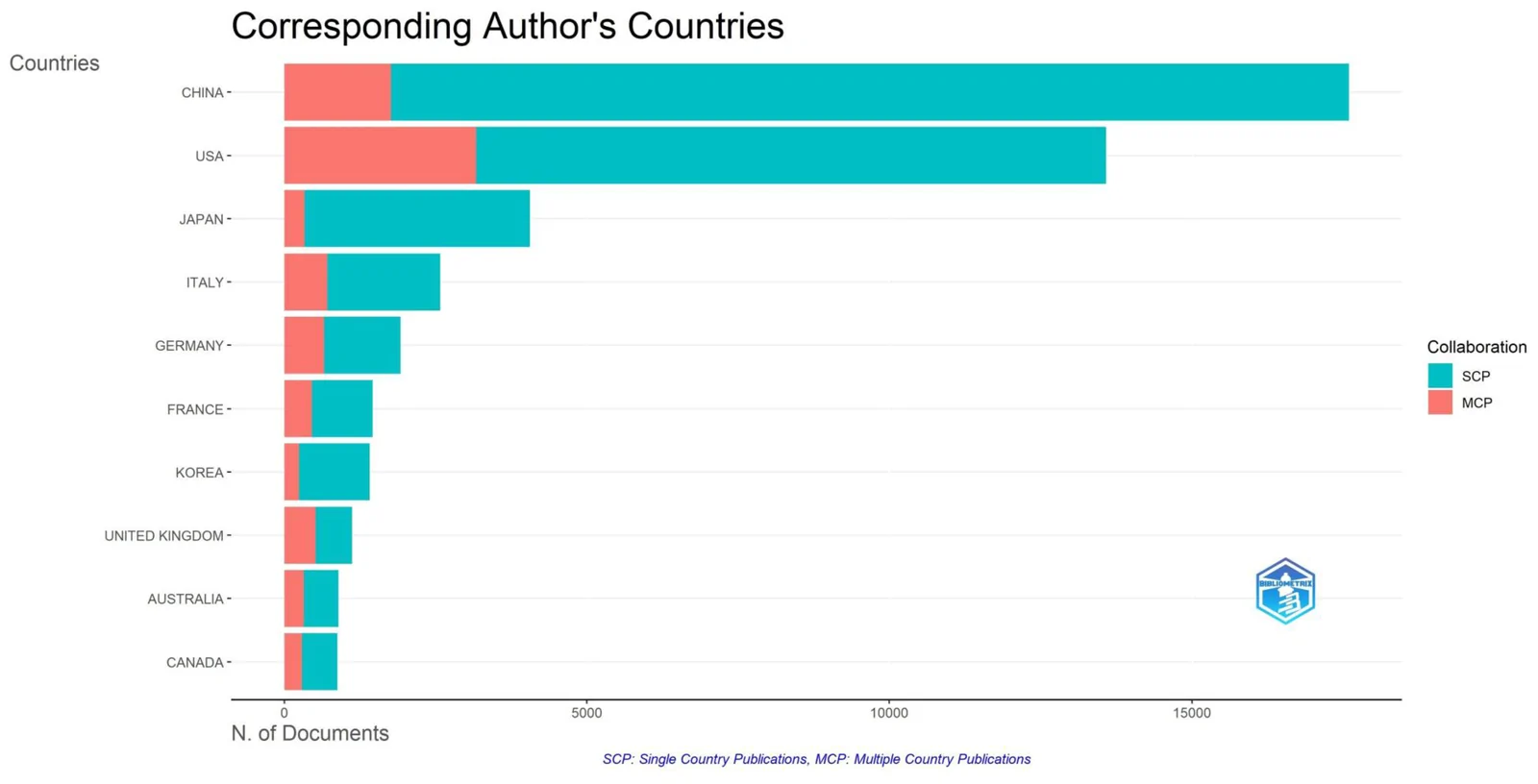
** Most productive countries based on corresponding author affiliations (2000-2025).** The bar chart distinguishes between Single Country Publications (SCP, blue) and Multiple Country Publications (MCP, red), highlighting differences in collaborative strategies between leading nations.

The analysis of corresponding authors' affiliations reveals a highly skewed geopolitical landscape dominated by two major powers, China and the USA, which together account for a substantial proportion of the total research output (Figure 3). China has emerged as the leading contributor, surpassing the USA in total publication volume. However, a deeper analysis of collaboration patterns (SCP vs. MCP) highlights distinct national strategies.

As shown in Figure 3, China's output is predominantly characterized by Single Country Publications (SCPs), indicating a strong internal research ecosystem but a relatively lower reliance on international partnerships than its total volume would suggest. In contrast, the USA demonstrates a higher proportion of Multiple Country Publications (MCP), suggesting a more integrated role in the global research network. Following this duopoly, Japan, Italy, and Germany round out the top five most productive nations, underscoring the significant contributions of both Asian and European clinical research hubs to the field.

To explicitly quantify these observational trends, country-level impact and collaboration metrics were analyzed. While China leads in absolute publication volume (17,593 articles) compared to the USA (13,582 articles), a quantitative divergence exists in per-article citation exposure and international collaboration intensity. The USA demonstrates a significantly higher average citation count per article, with an Average Article Citations (AAC) rate of 84.5 per document, compared to 22.7 for China. Furthermore, the analysis of collaboration ratios confirms the USA's extensive integration in international networks; 23.4% of the USA's research output consists of Multiple Country Publications (MCP), starkly contrasting with China's domestically oriented ecosystem, where the MCP ratio remains at 10.0%. These explicit metrics substantiate the observation that while publication volume has shifted toward Asia, US-affiliated research maintains both a higher per-article citation rate and a greater degree of international co-authorship.

### 3.3. Most Relevant Affiliations

**Figure 4:**
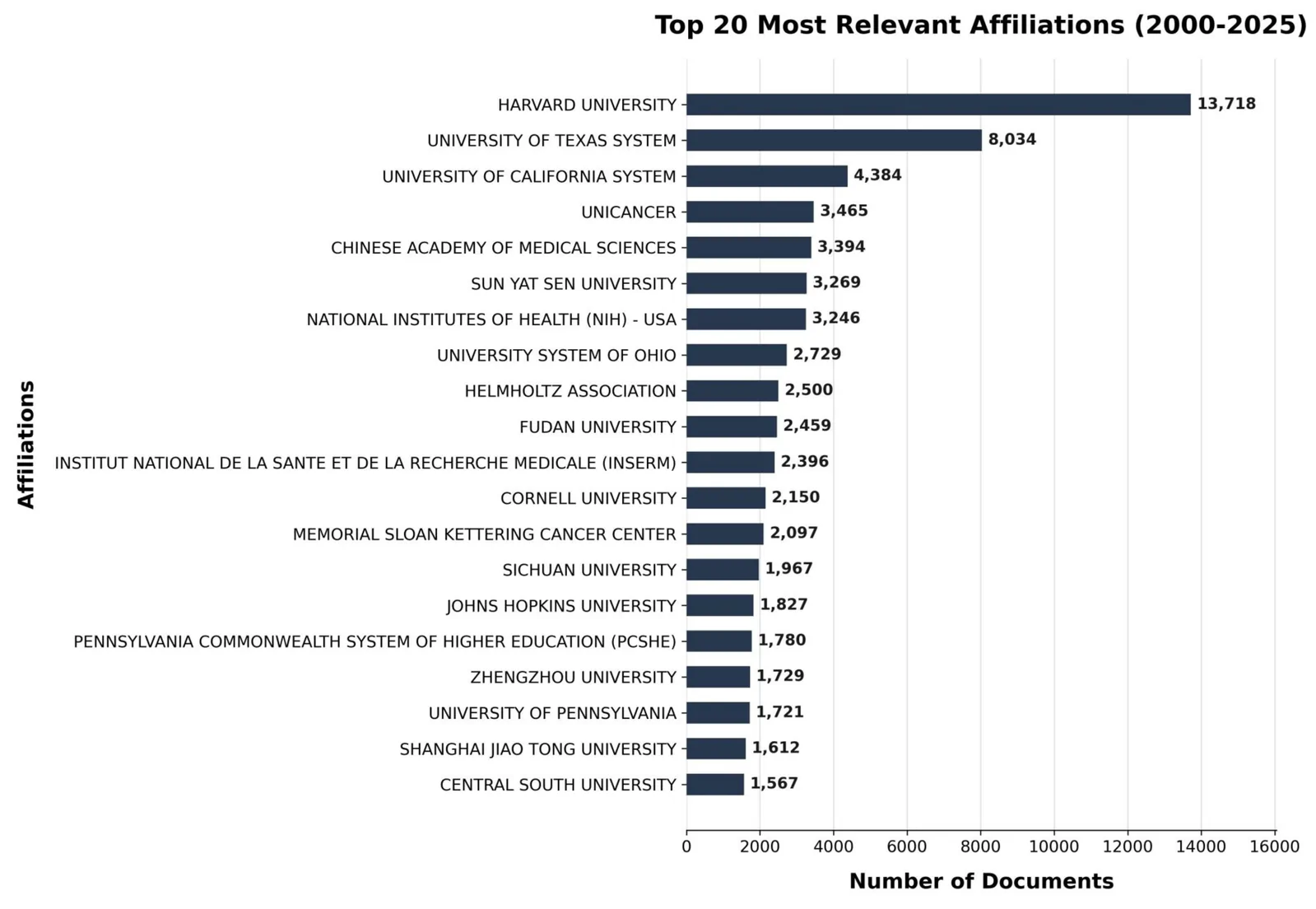
** Top 20 most relevant affiliations within the title-defined core literature of solid tumor immunotherapy (2000-2025).** The ranking illustrates the leadership of major US academic systems and the increasing prominence of Chinese universities.

An analysis of institutional contributions highlights the leading output of large American academic systems and the rapid ascent of Chinese research centers (Figure 4). Harvard University and its affiliated networks have emerged as the leading contributors to the field, with a massive, integrated research output. This is closely followed by the University of Texas System, driven largely by the specialized output of the MD Anderson Cancer Center, a global hub for immunotherapy trials.

Notably, Sun Yat-sen University (China) has secured a prominent position among the world's top global institutions, signaling a shift in the center of gravity in immuno-oncology research towards Asia. The list also features major European consortia such as Unicancer and INSERM (France), indicating a strong continental infrastructure for cancer research. The presence of historic pioneers such as Johns Hopkins University among the top 20 further underscores the sustained productivity of centers that laid the early groundwork for checkpoint blockade therapies.

### 3.4. Interconnections Between Countries, Topics, and Journals

**Figure 5:**
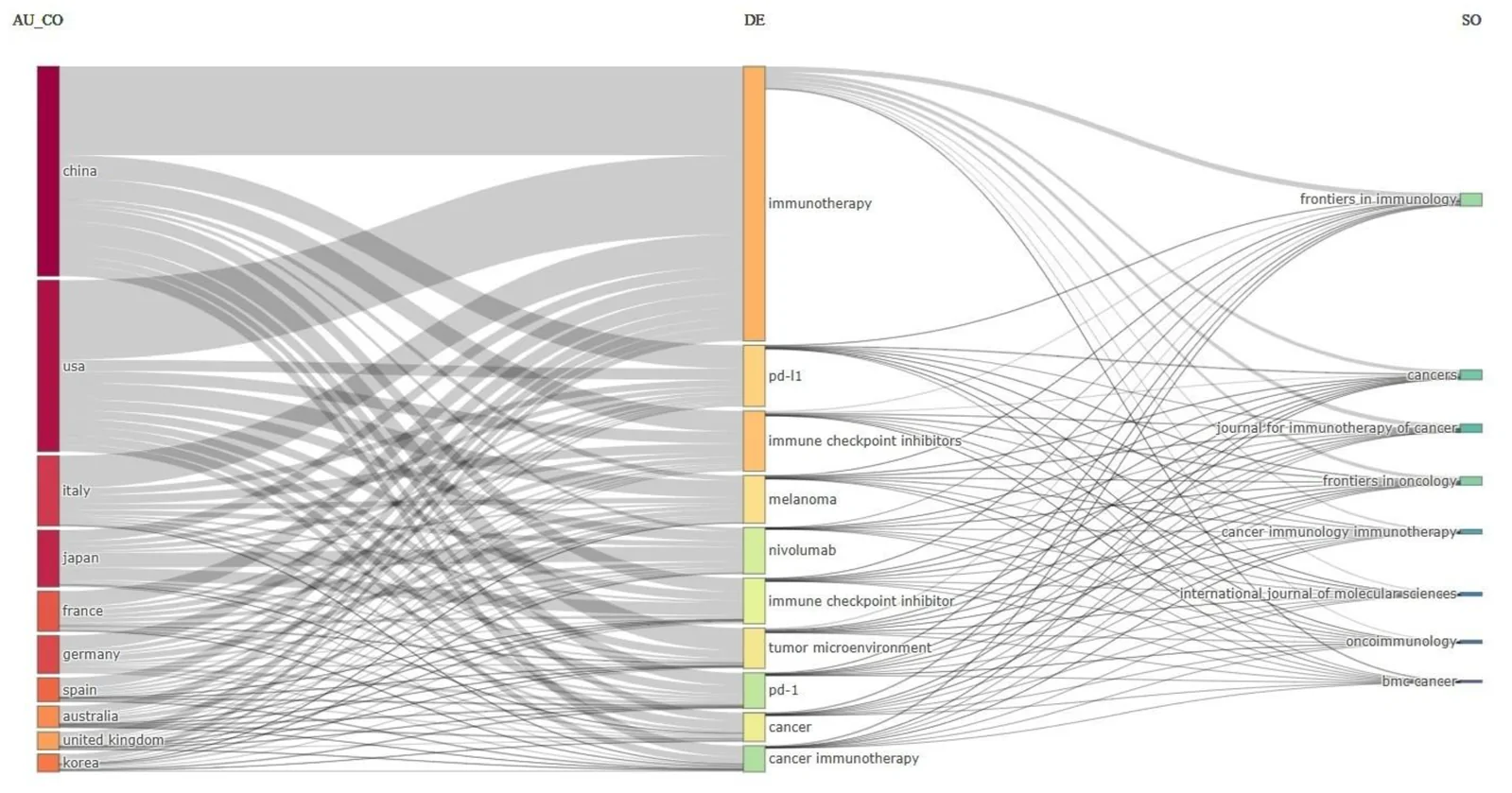
** Three-field plot (Sankey diagram) displaying the structural relationship between the most productive countries (left), author keywords (middle), and publication sources (right).** The width of the connecting nodes and gray lines is proportional to the number of publications. The diagram illustrates how research output from major contributors flows through specific thematic clusters into primary dissemination outlets. To ensure maximum readability and allow for detailed exploration of all nodes, a scalable, high-resolution version of this figure is provided in the Supplementary Material.

To visualize the dissemination of knowledge, a three-field plot (Sankey diagram) was constructed, linking the most productive countries (left), author keywords (middle), and target journals (right) (Figure 5).

The visualization reveals distinct publication preferences between the two major research hubs. China, currently the leading contributor in terms of volume, exhibits a strong preference for open-access platforms, with a significant flow of research directed towards leading open-access journals in the field. This trend aligns with the rapid expansion of Chinese research output and the increasing reliance on open-access models for global dissemination.

In contrast, the USA demonstrates a more diversified publication strategy. While also contributing significantly to open-access platforms like Frontiers in Immunology, American research flows are broadly distributed among specialized society journals such as the Journal for Immunotherapy of Cancer (JITC) and Cancer Immunology, Immunotherapy. The central keyword analysis confirms that while core topics, such as 'Immunotherapy' and 'PD-L1', are universal, dissemination channels vary by region, making high-impact open-access journals pivotal hubs for the international exchange of translational immuno-oncology data.

To systematically identify the field's core journals, Bradford's Law of Scattering was applied. The analysis identified a distinct 'Core Zone' comprising the most prolific sources. Frontiers in Immunology ranked first with the highest publication volume (2,751 articles), followed by Frontiers in Oncology, Cancers, and the Journal for Immunotherapy of Cancer (JITC). This empirical evidence underscores the prominent role of open-access platforms in disseminating immuno-oncology research, highlighting a global shift towards rapid and accessible scientific exchange in this domain.

### 3.5. Most Globally Cited Documents

The analysis of the most cited documents reveals that the field's intellectual foundation is built on landmark clinical trials and mechanistic reviews published in high-impact general medical journals (Table 3).

The most cited paper in the dataset is the pivotal phase 3 study by Hodi *et al*. (2010) 5 published in the New England Journal of Medicine (NEJM), which demonstrated the survival benefit of Ipilimumab in metastatic melanoma (12,142 citations). This is followed by Pardoll's (2012) 21 comprehensive review on the immunology of checkpoint blockade in Nature Reviews Cancer.

A striking finding is the overwhelming dominance of the NEJM, which published 9 of the top 10 most-cited papers (Table 3). This underscores the central role of pivotal clinical trials—such as those establishing the efficacy of Nivolumab (Topalian et al., 2012) 6, Pembrolizumab (Reck et al., 2016) 22, and Atezolizumab (Finn et al., 2020) 28—in driving the scientific impact and clinical transformation of the field.

### 3.6. Evolution of Research Themes

**Figure 6:**
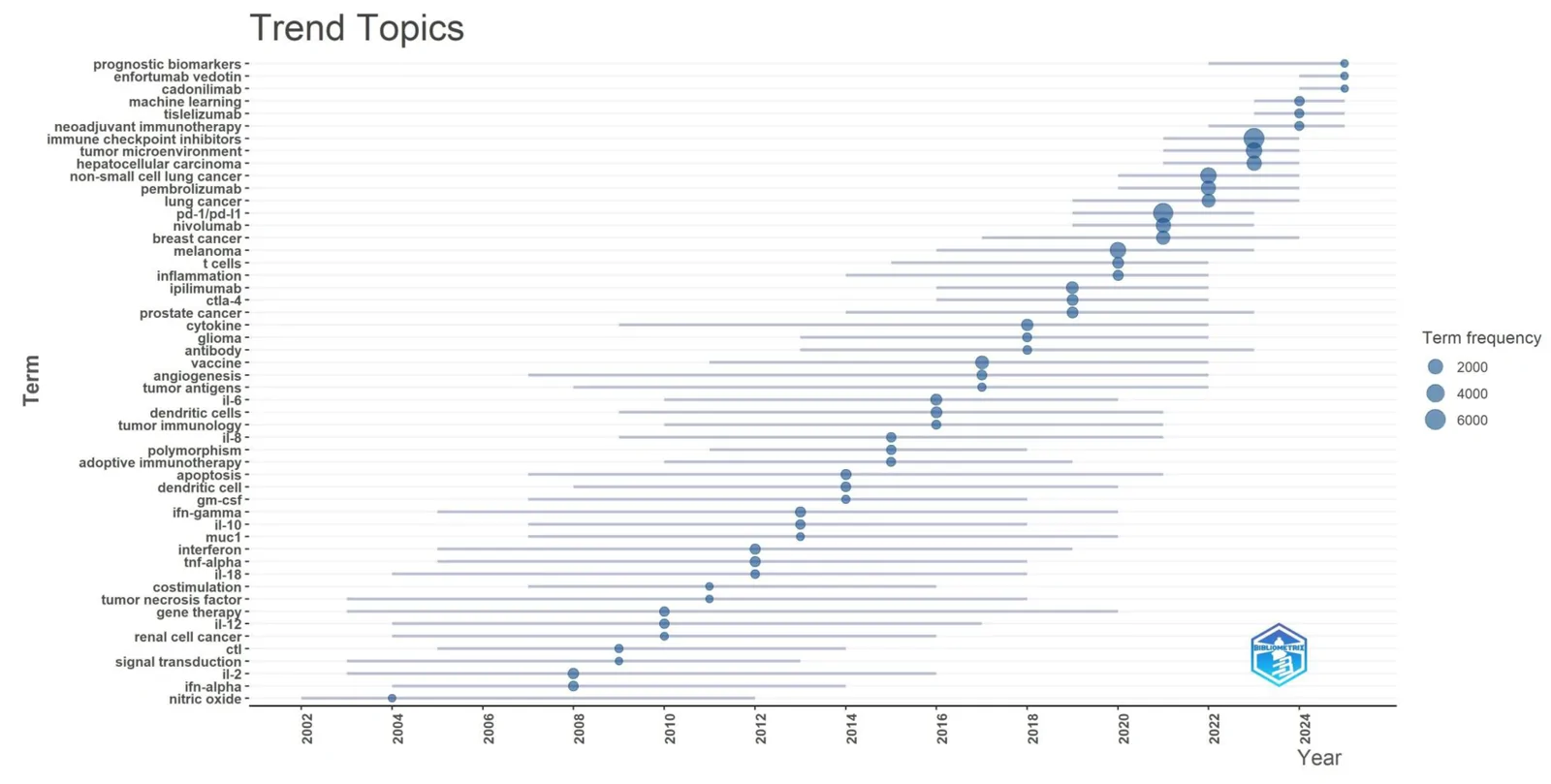
** Trend topics analysis of author keywords (2000-2025).** The visualization displays the temporal evolution of the most frequent research topics over the last quarter-century. The X-axis represents the timeline, while the Y-axis lists the keywords. The position of each bubble indicates the year of the median publication. Bubble size is proportional to the keyword's frequency. The blue horizontal lines represent the interquartile range (Q1-Q3), indicating the duration of each topic's prominence in the literature. To ensure maximum readability and allow for detailed exploration of all nodes, a scalable, high-resolution version of this figure is provided in the Supplementary Material.

To capture the quarter-century evolution of the field, a trend topic analysis was conducted spanning from the early 2000s to the present (Figure 6). The visualization reveals a dramatic paradigm shift in immuno-oncology research, characterized by three distinct eras:

**The Cytokine & Gene Therapy Era (2000-2010):** The foundational decade was dominated by non-specific immunomodulators. High-frequency keywords such as 'Interleukin-2 (IL-2)', 'Interferon-alpha', and 'Gene Therapy' define this period, reflecting early efforts to stimulate systemic anti-tumor immunity before the checkpoint revolution.

**The Checkpoint Revolution (2011-2019):** A fundamental paradigm shift occurred with the clinical success of checkpoint blockade. The research focus shifted predominantly to specific targets, with 'PD-L1', 'Nivolumab', and 'CTLA-4' becoming the central pillars of academic output. This era also saw significant interest in 'Dendritic Cells' and 'Vaccines', marking the transition from bench to bedside.

**The Era of Precision & ADCs (2020-2025):** In recent years, the research literature has pivoted towards greater complexity and the exploration of novel modalities. Emerging themes such as 'Tumor Microenvironment (TME)' and 'Neoadjuvant Immunotherapy' indicate a focus on mechanisms of resistance. Notably, the appearance of 'Machine Learning' and specific antibody-drug conjugates (ADCs) like 'Enfortumab Vedotin' highlights the growing investigational integration of computational tools and next-generation therapeutics into experimental and clinical trial frameworks.

### 3.7. Co-occurrence Network Visualization

**Figure 7:**
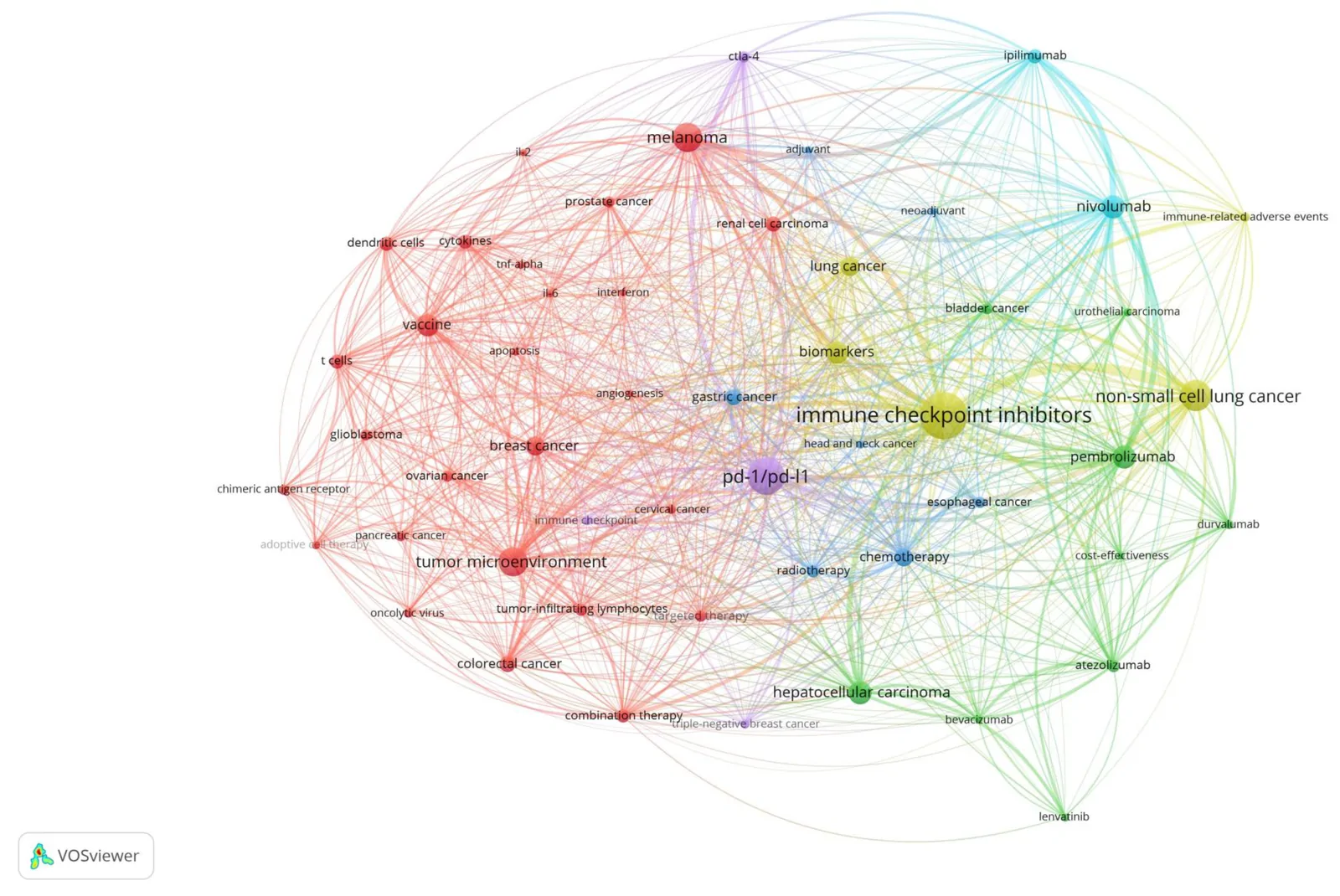
** Network visualization of keyword co-occurrence.** The size of the nodes is proportional to the frequency of the terms, while the distance between two nodes reflects the strength of their co-occurrence relationship. The colors represent distinct functional clusters: Red Cluster (Left): Biological mechanisms, Tumor Microenvironment (TME), and resistance pathways; Green Cluster (Right): Clinical standards of care, including Immune Checkpoint Inhibitors (ICIs) and major solid tumor indications (e.g., NSCLC); Blue Cluster (Top): Multimodal, perioperative, and neoadjuvant treatment strategies. Network parameters: Minimum keyword occurrence threshold = 250; Number of displayed nodes = 54; Normalization method = Association strength; Clustering resolution = 1.00. To ensure maximum readability and allow for detailed exploration of all nodes, a scalable, high-resolution version of this figure is provided in the Supplementary Material.

To map the field's conceptual topology, a keyword co-occurrence analysis was conducted (Figure 7). The network visualization reveals a structured research landscape divided into three primary functional domains:

**The Biological & Resistance Cluster (Left/Red):** This cluster is structurally dominated by 'Tumor Microenvironment (TME)' and 'Vaccine'. While it contains foundational mechanistic nodes, such as 'Cytokines', 'Angiogenesis' and 'Oncolytic Virus', the overwhelming density of the TME node suggests a major shift in research focus. The presence of 'Breast Cancer' and 'Ovarian Cancer' within this cluster suggests a growing research emphasis on investigating the manipulation of the microenvironment to potentially overcome resistance in non-immunogenic ('cold') tumors.

**The Clinical Standard & Checkpoint Cluster (Right & Center):** This extensive region represents the clinical implementation of immunotherapy. It is anchored by 'Immune Checkpoint Inhibitors' (Yellow-Green) and 'PD-1/PD-L1' (Central/Purple nodes), serving as the bridge between basic science and clinical application. The cluster radiates outward to specific agents, such as 'Nivolumab' (Cyan), and indications, including 'Non-Small Cell Lung Cancer (NSCLC)' and 'Hepatocellular Carcinoma' (Green), confirming that checkpoint blockade remains the central therapeutic pillar across diverse solid tumors.

**The Multimodal & Perioperative Cluster (Top/Blue**): A distinct grouping emerged around 'Chemotherapy', 'Adjuvant', and 'Neoadjuvant' strategies. The distinct positioning of the 'Neoadjuvant' term highlights a major focus in recent literature: the investigation of immunotherapy in earlier lines of treatment to evaluate its potential impact on pathological response rates.

### 3.8. Temporal Evolution of Research Themes

**Figure 8:**
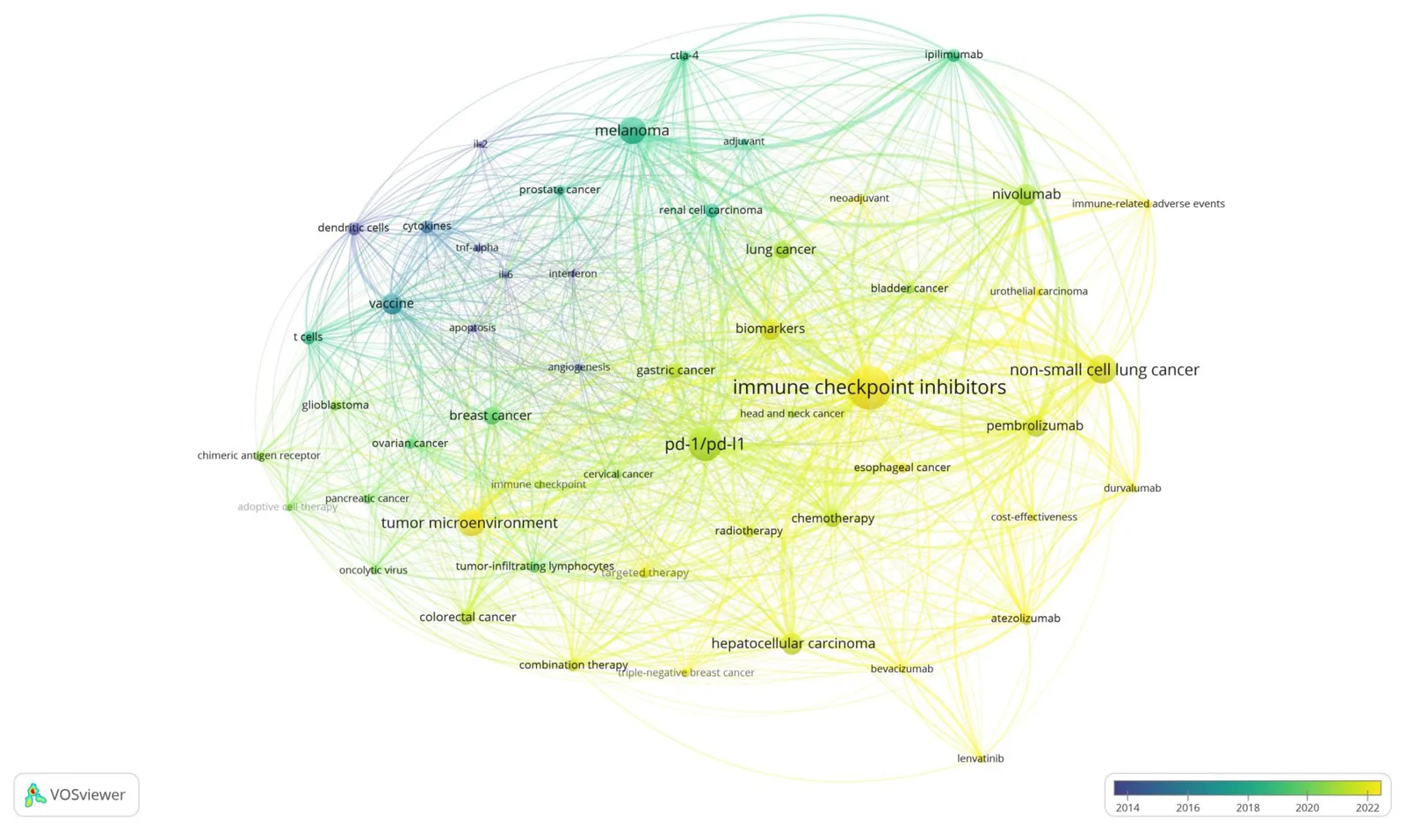
** Overlay visualization of keyword occurrences colored by average publication year.** The color scale represents the temporal evolution of research topics. Blue nodes indicate older, foundational themes (e.g., mechanisms such as apoptosis), while Yellow nodes represent the most recent and trending topics (e.g., neoadjuvant therapy, novel inhibitors), reflecting the growing research focus on advanced clinical applications. Network parameters: Minimum keyword occurrence threshold = 250; Number of displayed nodes = 54; Normalization method = Association strength; Clustering resolution = 1.00. To ensure maximum readability and allow for detailed exploration of all nodes, a scalable, high-resolution version of this figure is provided in the Supplementary Material.

To trace the field's strategic evolution, a temporal overlay analysis was applied to the co-occurrence network (Figure 8). The color gradient from blue (older) to yellow (newer) reveals a decisive shift in research priorities:

**Historical Foundations (Blue/Purple - 2010-2016):** The earliest phase of research was dominated by fundamental immunobiology. Terms such as 'Cytokines', 'Dendritic Cells', and 'Apoptosis' appear in dark blue, indicating that the initial decade was dedicated to understanding the basic mechanisms of immune activation before the widespread clinical success of checkpoint inhibitors.

**The Modern Investigational Era (Yellow/Green - 2021-2025):** The research landscape has recently pivoted towards advanced clinical applications. 'Lenvatinib' (Avg. Year 2023.1) and 'Neoadjuvant' (Avg. Year 2022.5) emerge as the most current hotspots. This highlights a marked increase in research activity investigating complex combinatorial strategies (e.g., Lenvatinib + ICI) and the potential integration of immunotherapy into early-stage treatment settings (neoadjuvant protocols), particularly in challenging malignancies like 'Triple-Negative Breast Cancer'.

### 3.9. Thematic Evolution and Strategic Diagrams

**Figure 9:**
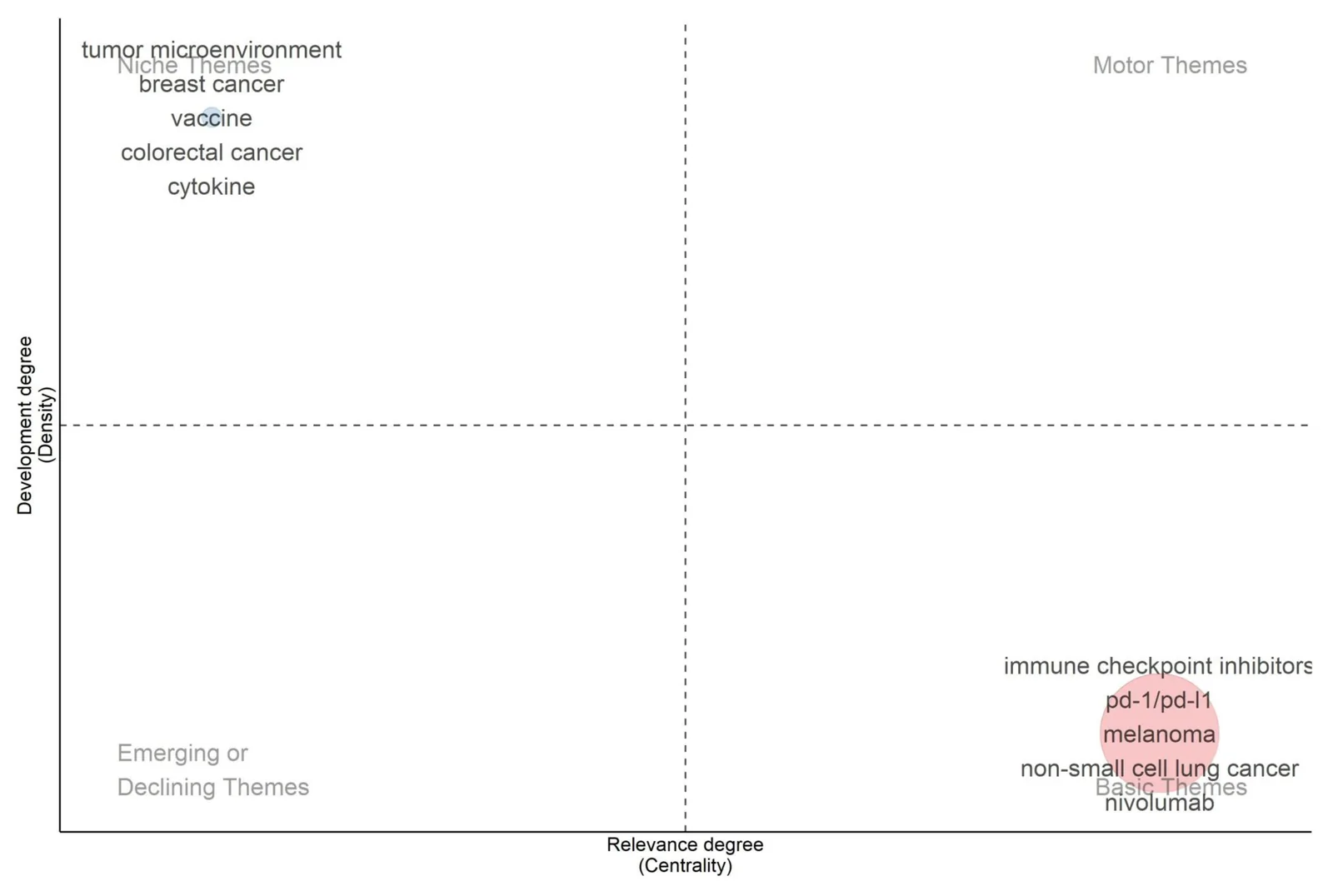
** Thematic Map of the core research landscape (2000-2025).** The diagram illustrates the conceptual structure of the core literature based on keyword co-occurrence clusters. The X-axis represents Centrality (relevance to the core research landscape), while the Y-axis represents Density (internal development of the topic). The analysis reveals a polarized landscape dominated by Basic Themes (lower right) representing foundational knowledge and Niche Themes (upper left) representing highly specialized areas of investigation.

To evaluate the conceptual maturity and strategic direction of the field, a Thematic Map was generated based on Callon's centrality and density measures (Figure 9). The visualization reveals a distinct polarization in the research landscape, characterizing immuno-oncology as a highly mature field with established foundations and specialized advanced research foci:

**Basic & Transversal Themes (Lower-Right Quadrant):** The cluster containing 'Immune Checkpoint Inhibitors', 'PD-1/PD-L1', and 'Melanoma' is situated in the quadrant of high centrality. This positioning identifies these topics as the domain's foundational pillars. Unlike "Motor Themes" which are still driving rapid change, these topics have evolved into the essential bedrock of modern oncology literature, indicating their consolidation as foundational research domains rather than experimental niches.

**Niche & Highly Developed Themes (Upper-Left Quadrant):** Conversely, the cluster comprising 'Tumor Microenvironment (TME)', 'Breast Cancer', and 'Vaccines' appears in the niche quadrant (high density, lower centrality). This suggests that research has shifted from general principles to highly specialized applications. The scientific community is now conducting deep, mechanistic investigations in specific contexts—such as overcoming resistance in "cold" tumors like breast cancer—rather than generic immunotherapy trials.

### 3.10. Comparative Trends in the Scopus High-Impact Subset

A parallel analysis of the 20,000 most-cited documents in Scopus was evaluated to observe the stability of core thematic clusters. While direct quantitative comparison is precluded by the vast difference in initial dataset sizes and the inherent citation-age bias of a highly-cited subset, qualitative observation revealed structural congruence in the field's foundational architecture.

The comparative subset analysis yielded the following key consistencies:

**Global Contribution:** Consistent with the WoSCC primary data, the high-impact Scopus subset highlighted the leading combined contribution of China and the USA in publication volume Supplementary Figure 1B).

**Journal Productivity:** Specialized immunology and oncology journals accounted for the majority of publications within this high-impact subset (Supplementary Figure 1C).

**Thematic Consistency:** VOSviewer keyword co-occurrence analysis of this highly cited subset (Supplementary Figure 1D) revealed a thematic pattern broadly consistent with the major historical domains identified in the primary WoSCC network. The map highlighted three distinct research pillars highly congruent with the primary WoSCC network: The Tumor Microenvironment and Prognostic Biomarkers (Cluster 1), centered on terms such as "Tumor microenvironment" and "Biomarkers" linked to major solid tumors; Inflammatory Mechanisms and Oxidative Stress (Cluster 2), focused on fundamental drivers such as "Cytokines" and "Ferroptosis"; and Clinical Checkpoint Blockade (Cluster 3), defined by the strong association between "Immune checkpoint inhibitors" and "PD-1/PD-L1".

This observation is consistent with the finding that the foundational thematic architecture of solid-tumor immuno-oncology remains robust across different database scopes, even when accounting for coverage disparities.

## 4. DISCUSSION

The trajectory of cancer immunotherapy over the last quarter-century represents one of the most remarkable narrative arcs in modern medicine. Our bibliometric analysis of the core literature reveals a landscape that has transitioned in parallel with the field's clinical evolution, from an era of skepticism and limited efficacy (2000-2010) to becoming the indisputable 'fourth pillar' of oncological care, alongside surgery, chemotherapy, and radiotherapy (2,3. The rapid descriptive expansion of the literature identified in our longitudinal data (Figure 2)—specifically the substantial surge in scientific production following 2011—chronologically mirrors the true clinical paradigm shifts driven by the validation of CTLA-4 and PD-1 blockade. This acceleration was not merely quantitative but represented a qualitative legitimization of the hypothesis that the host immune system could be harnessed to achieve durable remissions. Landmark studies by Hodi et al. 5 and Topalian et al. 6 provided the foundational clinical evidence, demonstrating unprecedented durable responses in metastatic melanoma and non-small cell lung cancer (NSCLC). This momentum was definitively cemented by the 2018 Nobel Prize in Physiology or Medicine 8, which validated the therapeutic potential of negative immune regulation and accelerated the transition from 'proof-of-concept' trials to global standard-of-care implementation.

Beyond the temporal evolution, our analysis illuminates a major quantitative redistribution in the geopolitical landscape of immuno-oncology. While the United States has historically served as the central node of innovation — demonstrating the highest proportion of international (multi-country) co-authorship among the leading producing nations (Figure 3) and hosting the most influential institutions (Figure 4) — the rapid ascent of China challenges this historical concentration of publication volume. Our data indicate that China currently exhibits the highest total publication output, a trend coinciding with substantial national investments in pharmaceutical research and the rapid expansion of domestic clinical trials. Recent industry reports confirm that China now accounts for a significant portion of the global oncology pipeline 29. Interestingly, this geographical expansion coincides with a decisive shift in dissemination channels; our data identifies high-impact open-access platforms as the primary conduit for this rapidly growing body of Asian-Pacific research. However, a critical divergence in publication and clinical strategy remains evident. Comparative analyses of trial registries reveal that while China has accelerated late-stage (Phase III) domestic trials, the US retains a substantial lead in multi-country, global clinical investigations (47.6% vs 2.2%) 16. This distinction aligns with our bibliometric finding that China's output is heavily characterized by Single Country Publications (SCP). Consequently, while China has achieved quantitative leadership in raw publication volume, the US maintains a leading role in international cross-pollination and cross-border collaborative networks 16,17,29. It should be noted that all institutional and country-level rankings are based on full-counting methods, with institutional affiliations systematically harmonized at the parent-organization level (Methods, Section 2.4) to prevent the artificial fragmentation of hospital, cancer center, and medical school output from their parent university systems, thereby ensuring data integrity.

The conceptual evolution of the field, as visualized through our network analyses (Figure 7 and Figure 9), mirrors the maturation of our biological understanding. The thematic map confirms that immune checkpoint inhibitors (ICIs) have evolved from 'emerging trends' to 'basic themes,' becoming a fundamental cornerstone of the modern oncological research landscape—widely investigated and foundational. However, the most striking finding is the structural prominence of the 'Tumor Microenvironment (TME)' cluster in recent years. This signals a directional shift in research focus: following the clinical validation of ICIs in 'hot' immunogenic tumors like melanoma and lung cancer, the bibliometric data show the scientific community is increasingly focusing its publication efforts on the complex challenge of 'cold' tumors. The strong co-occurrence of terms such as 'resistance,' 'TME,' and 'reprogramming' reflects a prevailing scientific hypothesis in the literature that overcoming the immunosuppressive barriers of the microenvironment may require combinatorial strategies rather than single checkpoint molecules 30,31. This aligns with recent comprehensive reviews suggesting that 'TME reprogramming'—through the integration of metabolic modulation, stromal targeting, and novel combinations—is widely discussed in the literature as an emerging strategy to potentially extend the benefits of immunotherapy to non-responders 30,32. However, it is crucial to explicitly delineate bibliometric observations from clinical implementation. The rapid expansion and high network visibility of nodes such as "tumor microenvironment", "triple-negative breast cancer", and "antibody-drug conjugates" clearly demonstrate that these domains are the primary targets of academic and translational prioritization. Yet, high publication volume alone cannot establish a therapeutic sequence or validate clinical efficacy. As modern precision oncology evolves, defining true clinical priorities requires triangulating bibliometric trends with layered molecular profiling, biomarker validation, mechanistic resistance data, and, ultimately, definitive trial evidence 11,33. Therefore, the high structural prominence of these topics in our co-occurrence networks should be interpreted as the scientific community's collective hypothesis-generation phase, which currently awaits broad translation into standard-of-care guidelines.

While this study provides a comprehensive mapping of the title-defined core literature in the solid tumor immunotherapy landscape, several limitations inherent to bibliometric analyses should be acknowledged. First, the Web of Science Core Collection (WoSCC) was utilized as the primary data source. Although we mitigated the potential coverage bias of using a single database by performing a parallel comparative subset analysis with the top 20,000 most-cited documents from Scopus, publications indexed exclusively in other niche databases (e.g., Embase or regional repositories) might still have been excluded. Second, the analysis was limited to publications in English, which is the lingua franca of medical science. This criterion, although necessary for accurate keyword co-occurrence analysis, may have led to the exclusion of relevant studies published in languages other than English, particularly from non-English-speaking regions with significant research output. Third, our search strategy was intentionally restricted to the "Title" (TI) field to maximize precision. We acknowledge this as a significant limitation, representing a deliberate trade-off that sacrifices search sensitivity (recall) in favor of specificity. Consequently, this study maps the "core, title-defined subset" of solid-tumor immunotherapy rather than the entire field. Many highly relevant mechanistic, computational, and biomarker studies that do not utilize generic immunotherapy terms or specific drug names in their titles are inevitably underrepresented in this dataset. Conversely, a title-centric strategy inherently overrepresents clinical trials and direct therapeutic interventions. While this limitation means our dataset is not entirely exhaustive, it ensures that the resulting thematic maps remain strictly focused on definitive immuno-oncology research free from the dilutive noise of incidental abstract mentions. Therefore, a TI-restricted search represents a deliberate and necessary methodological choice to ensure high-fidelity thematic mapping. While this approach ensures a high-fidelity dataset, it is possible that some relevant studies with non-descriptive titles may have been overlooked. Fourth, to visualize macro-topological paradigm shifts across 54,546 documents, our keyword harmonization strategy deliberately aggregated specific biological subsets (e.g., regulatory T cells into general T cells) and distinct targets (e.g., anti-PD-1 into immune checkpoint inhibitors). We acknowledge that while this aggressive clustering effectively illustrates overarching clinical and historical trends, it inherently sacrifices micro-level biological resolution, thereby masking the intricate, often opposing molecular dynamics within specific domains, such as the tumor microenvironment. Finally, bibliometric metrics such as citation counts are quantitative indicators of scientific impact and interest but do not necessarily reflect the intrinsic scientific quality or clinical validity of individual studies. Furthermore, citation analysis for the most recent years (2024-2025) should be interpreted with caution, as these publications have had less time to accumulate citations than older landmark studies, a phenomenon known as "time-lag bias"^34^. In alignment with the principles of the Leiden Manifesto for research metrics 35, we must explicitly state that the citation indicators reported herein are based on raw, unnormalized counts rather than field- or year-normalized metrics. Consequently, raw citation volumes intrinsically favor older foundational studies and large-scale Phase III trials due to their extended exposure windows. Therefore, any citation-based comparisons of geopolitics or institutions should be interpreted strictly as an index of absolute historical impact, rather than as a normalized measure of current scientific quality. Despite these limitations, the trends identified in this quarter-century analysis, and structurally observed across both database subsets, provide a robust and representative overview of the field's evolution. Ultimately, our analysis evaluates the absolute volume of solid-tumor immunotherapy publications rather than a normalized ratio against the total global oncology literature. As such, the observed rapid growth trajectory must be interpreted with caution; a proportion of this expansion inevitably reflects broader systemic factors, including the global proliferation of open-access publishing models, continuous database expansion, and the overall growth of general medical research output. Additionally, the 2025 publication metrics should be regarded as near-final estimates, given the inherent database indexing lag preceding our extraction date of January 1, 2026.

As we stand at the threshold of the next quarter-century, our temporal analysis (Figure 8) provides a hypothesis-generating interpretation of current research priorities, supported by bibliometric visibility. The prominent emergence of 'Neoadjuvant' therapies (Avg. Year 2022.5) and 'Triple-Negative Breast Cancer' as the most recent hotspots is consistent with a growing academic interest in investigational early-stage settings, a hypothesis further supported by the recent regulatory approvals of neoadjuvant immunotherapy regimens. Importantly, this identified research trajectory is not derived solely from keyword density but aligns with recent external regulatory approvals and paradigm-altering Phase III trials—most notably CheckMate-816 in NSCLC and KEYNOTE-522 in early TNBC 36,37. This publication trend reflects the biological rationale that administering immunotherapy in the perioperative setting may leverage a more intact host immune system to improve pathological complete response (pCR) rates before surgical resection 38. Ultimately, our study portrays a field in constant methodological and clinical evolution: moving from the non-specific cytokine storms of the past to the precision checkpoint blockade of the present, and currently highlighting strong investigational interest in multimodal synergy. Recent publication trajectories suggest growing academic interest in evaluating combinatorial strategies—particularly the conceptual combinations of ICIs with synergistic targeted agents, such as antibody-drug conjugates (ADCs). While ADCs are strictly distinct from classical immunotherapies, their high visibility in recent bibliometric networks reflects robust academic interest in their hypothesized potential as combinatorial partners to induce immunogenic cell death (ICD) and overcome resistance in historically non-immunogenic malignancies 39.

## 5. CONCLUSION

This comprehensive bibliometric analysis encapsulates the twenty-five-year structural maturation of the core, title-defined literature surrounding solid tumor immunotherapy from an experimental niche to a fundamental pillar of oncology. By mapping the trajectory from the cytokine era to the checkpoint revolution and the current neoadjuvant shift, our study provides a high-resolution roadmap of this core research landscape. These data-driven insights provide a comprehensive historical overview and serve as a hypothesis-generating resource for researchers navigating the complex and rapidly evolving era of combinatorial precision medicine.

## Supplementary Material

Supplementary figures and tables.

High resolution figures.

## Figures and Tables

**Table 1:** Methodological and conceptual comparison of prior bibliometric reviews versus the present study.

Representative Reference	Time Period	Database & Search Field	Sample Size (Documents)	Tumor & Modality Scope	Main Focus / Limitations	Unique Contribution of the Present Study
Wu *et al*., 2025(14)	2004-2024	WoSCC (N/A)	662	Restricted to Diffuse Large B-cell Lymphoma	Analyzes hematological immunotherapy, which is fundamentally distinct from solid tumor microenvironments	Solid Tumor Specificity: Exclusively isolates solid tumor dynamics
Hua *et al*., 2025(15)	2004-2024	WoSCC (Topic/TS)	780	Restricted to a single solid malignancy (Esophageal Cancer)	Captures organ-specific trends but fails to illustrate the global cross-pollination of immunological knowledge across different oncology disciplines.	Pan-Tumor Cross-Pollination: Maps overarching biological trends across all solid tumors.
Shi *et al*., 2025(16)	2014-2024	NMPA (N/A) and ClinicalTrials.gov (N/A)	846	Restricted to Cancer Vaccines in China and the USA	Modality-restricted (only vaccines) and geographically confined; misses the broader ICI and combinatorial landscape.	Pan-Modality & Global: Evaluates all immunotherapy classes on a truly global scale.
Zhang *et al*., 2025(17)	2015-2024	WoSCC (Topic/TS)	11,128	Restricted to Tumor Immune Escape	Mechanistic focus is restricted to a specific biological phenomenon rather than the broader clinical/therapeutic landscape.	Clinical Translation: Maps the complete clinical evolution.
Current Manuscript	2000-2025	WoSCC (Title/TI)	54,546	All solid malignancies; comprehensive systemic immuno-oncology agents.	Macro-level topological mapping restricted to clinical and translational solid tumor data.	Topological Era Mapping: Delivers the quarter-century structural evolution map.

**Table 2:** Main information of the dataset (2000-2025)

Description	Results
MAIN INFORMATION Timespan Sources (Journals, Books, etc.) Documents Annual Growth Rate % Document Average Age Average citations per doc	2000:2025 1563 54546 11.26 6.53 44.51
DOCUMENT CONTENTS Keywords Plus (ID) Author's Keywords (DE)	28219 41409
AUTHORS Authors Authors of single-authored docs	161651 799
AUTHORS COLLABORATION Single-authored docs Co-Authors per Doc International co-authorships %	1009 9.06 20.87

Note: Annual growth rate as calculated by Biblioshiny (CAGR); log-linear OLS estimate reported in Results section.

**Table 3:** Top 10 most globally cited documents in immuno-oncology research.

Rank	Paper (Author, Year, Journal)	Total Citations (TC)	TC per Year
1	Hodi FS, 2010, N Engl J Med(5)	12142	714.24
2	Pardoll DM, 2012, Nat Rev Cancer(21)	11019	734.60
3	Topalian SL, 2012, N Engl J Med(6)	10298	686.53
4	Reck M, 2016, N Engl J Med(22)	8144	740.36
5	Borghaei H, 2015, N Engl J Med(23)	7873	656.08
6	Le DT, 2015, N Engl J Med(24)	7475	622.92
7	Brahmer JR, 2012, N Engl J Med(25)	6545	436.33
8	Larkin J, 2015, N Engl J Med(26)	6092	507.67
9	Brahmer J, 2015, N Engl J Med(27)	5838	486.50
10	Finn RS, 2020, N Engl J Med(28)	5478	782.57

## Data Availability

In accordance with the licensing terms of Clarivate Analytics (Web of Science) and Elsevier (Scopus), the raw bibliographic data exports and the associated Biblioshiny R workspace file cannot be publicly redistributed. These materials are available from the corresponding author upon reasonable request, subject to the requester's compliance with the relevant database licensing terms. To ensure full methodological transparency and computational reproducibility, all components within our ownership or license are publicly available as supplementary materials accompanying this manuscript. These include: (i) the complete, copy-pasteable search strings for both databases (Supplementary Table S1); and (ii) the custom VOSviewer thesaurus harmonization file, which encodes all synonym-merging applied before network generation. No custom R scripts were generated, as all primary bibliometric analyses were performed interactively via the Biblioshiny graphical user interface. All additional analytical parameters sufficient for full replication —including software versions (bibliometrix v5.2.1, R v4.5.2, VOSviewer v1.6.20), minimum keyword occurrence thresholds, network normalization methods, and clustering resolution settings— are explicitly documented within the Methods section and the respective figure legends.

## Abbreviations

AAC: Average Article Citations
ADC: antibody-drug conjugate
AI: artificial intelligence
BiTE: bispecific T-cell engager
CAGR: compound annual growth rate
CAR: chimeric antigen receptor
CTLA-4: cytotoxic T-lymphocyte-associated protein 4
EMA: European Medicines Agency
FDA: Food and Drug Administration
ICD: immunogenic cell death
ICI: immune checkpoint inhibitor
JITC: Journal for Immunotherapy of Cancer
MCP: Multiple Country Publications
NEJM: New England Journal of Medicine
NMPA: National Medical Products Administration
NSCLC: non-small cell lung cancer
OLS: Ordinary Least Squares
PD-1: programmed cell death protein 1
PD-L1: programmed death-ligand 1
PMDA: Pharmaceuticals and Medical Devices Agency
SCP: Single Country Publications
TCR: T-cell receptor
TIL: tumor-infiltrating lymphocyte
TME: tumor microenvironment
TNBC: triple-negative breast cancer
WoSCC: Web of Science Core Collection
