# Supplementary material for: Mapping the Evolutionary Landscape of Solid Tumor Immunotherapy: A Quarter-Century Bibliometric Analysis of the Title-Defined Core Literature (2000-2025)
Source: Int J Med Sci. 2026 Jul 22;23(9):2810–27. doi: 10.7150/ijms.137251 (PMC13539906; doi:10.7150/ijms.137251)
Supplement: Supplementary file 1 [file ijmsv23p2810s1.pdf]

Supplementary Table S1.

PANEL A: Detailed search strategy, query strings, and filtering criteria used for data retrieval from the Web of Science Core Collection (WoSCC).

Search Set	Description	Search Query (Field Tag: TI = Title)
#1	**Immunotherapy & Agents**	("immunotherap*" OR "immuno-oncolog*" OR "immune checkpoint inhibitor*" OR "checkpoint blockade" OR "PD-1" OR "PD-L1" OR "CTLA-4" OR "LAG-3" OR "TIGIT" OR "TIM-3" OR "nivolumab" OR "pembrolizumab" OR "cemiplimab" OR "dostarlimab" OR "atezolizumab" OR "durvalumab" OR "avelumab" OR "ipilimumab" OR "tremelimumab" OR "relatlimab" OR "tiragolumab" OR "botensilimab" OR "camrelizumab" OR "sintilimab" OR "toripalimab" OR "tislelizumab" OR "penpulimab" OR "zimberelimab" OR "serplulimab" OR "sugemalimab" OR "envafolimab" OR "prolgolimab" OR "balstilimab" OR "retifanlimab" OR "cadonilimab" OR "ivonescimab" OR "CAR-T" OR "CAR T-cell*" OR "chimeric antigen receptor*" OR "CAR-NK*" OR "NK cell therap*" OR "TIL therapy" OR "tumor infiltrating lymphocyte*" OR "lifileucel" OR "TCR-T" OR "adoptive cell therap*" OR "oncolytic virus*" OR "oncolytic virotherap*" OR "T-VEC" OR "talimogene" OR "interleukin*" OR "cytokine therap*" OR "interferon*" OR "cancer vaccin*" OR "tumor vaccin*" OR "neoantigen vaccin*" OR "sipuleucel-T" OR "bispecific antibod*" OR "BiTE" OR "T-cell engager*" OR "tarlatamab" OR "amivantamab" OR "tebentafusp" OR "immune agonist*" OR "immune modulat*" OR "STING agonist*")
#2	**Solid Tumors Inclusion**	("cancer*" OR "oncolog*" OR "tumor*" OR "neoplasm*" OR "carcinoma*" OR "malignanc*" OR "sarcoma*" OR "melanoma*" OR "glioblastoma*" OR "neuroblastoma*" OR "mesothelioma*" OR "metastasis*" OR "metastatic")
#3	**Hematology Exclusion**	("leukemia*" OR "lymphoma*" OR "myeloma*" OR "hematologic*")

Search Set	Description	Search Query (Field Tag: TI = Title)
**#4**	**Final Combined Strategy**	**(#1 AND #2) NOT #3**
**#5**	**Filters & Refinements**	**Indices:** SCI-EXPANDED, ESCI.<br><br>**Document Types:** Article, Review.<br><br>**Language:** English.<br><br>**Time Span:** 2000–2025.<br><br>**Research Areas:** Oncology; Immunology; Pharmacology Pharmacy; Cell Biology; Medicine Research Experimental; Biochemistry Molecular Biology; Medicine General Internal.

Note: TI denotes "Title Search". This strategy ensures high specificity by retrieving records where the keywords appear primarily in the title of the manuscript.

PANEL B: Detailed search strategy, query strings, and filtering criteria used for data retrieval from the Scopus database.

Search Set	Description	Search Query (Field Tag: TITLE-ABS-KEY)
**#1**	**Immunotherapy & Agents**	("immunotherap*" OR "immuno-oncolog*" OR "immune checkpoint inhibitor*" OR "checkpoint blockade" OR "PD-1" OR "PD-L1" OR "CTLA-4" OR "LAG-3" OR "TIGIT" OR "TIM-3" OR "nivolumab" OR "pembrolizumab" OR "cemiplimab" OR "dostarlimab" OR "atezolizumab" OR "durvalumab" OR "avelumab" OR "ipilimumab" OR "tremelimumab" OR "relatlimab" OR "tiragolumab" OR "botensilimab" OR "camrelizumab" OR "sintilimab" OR "toripalimab" OR "tislelizumab" OR "penpulimab" OR "zimberelimab" OR "serplulimab" OR "sugemalimab" OR

Search Set	Description	Search Query (Field Tag: TITLE-ABS-KEY)
		"envafolimab" OR "prolgolimab" OR "balstilimab" OR "retifanlimab" OR "cadonilimab" OR "ivonescimab" OR "CAR-T" OR "CAR T-cell*" OR "chimeric antigen receptor*" OR "CAR-NK*" OR "NK cell therap*" OR "TIL therapy" OR "tumor infiltrating lymphocyte*" OR "lifileucel" OR "TCR-T" OR "adoptive cell therap*" OR "oncolytic virus*" OR "oncolytic virotherap*" OR "T-VEC" OR "talimogene" OR "interleukin*" OR "cytokine therap*" OR "interferon*" OR "cancer vaccin*" OR "tumor vaccin*" OR "neoantigen vaccin*" OR "sipuleucel-T" OR "bispecific antibod*" OR "BiTE" OR "T-cell engager*" OR "tarlatamab" OR "amivantamab" OR "tebentafusp" OR "immune agonist*" OR "immune modulat*" OR "STING agonist*")
#2	**Solid Tumors Inclusion**	("cancer*" OR "oncolog*" OR "tumor*" OR "neoplasm*" OR "carcinoma*" OR "malignanc*" OR "sarcoma*" OR "melanoma*" OR "glioblastoma*" OR "neuroblastoma*" OR "mesothelioma*" OR "metastasis*" OR "metastatic")
#3	**Hematology Exclusion**	("leukemia*" OR "lymphoma*" OR "myeloma*" OR "hematologic*")
#4	**Final Combined Strategy**	**(#1 AND #2) NOT #3**
#5	**Filters & Refinements**	**Source Type:** Journal<br>**Document Types:** Article, Review.<br>**Language:** English.<br>**PUBYEAR:** >1999 AND <2026.

Search Set	Description	Search Query (Field Tag: TITLE-ABS-KEY)
		**Subject Areas:** Medicine; Biochemistry, Genetics and Molecular Biology; Immunology and Microbiology; Pharmacology, Toxicology and Pharmaceutics

Note: TITLE-ABS-KEY denotes "Article title, Abstract, Keywords". This strategy aims to test whether global structural patterns are stable across databases.

Final String for both WoSCC and Scopus.:

("immunotherap*" OR "immuno-oncolog*" OR "immune checkpoint inhibitor*" OR "checkpoint blockade" OR "PD-1" OR "PD-L1" OR "CTLA-4" OR "LAG-3" OR "TIGIT" OR "TIM-3" OR "nivolumab" OR "pembrolizumab" OR "cemiplimab" OR "dostarlimab" OR "atezolizumab" OR "durvalumab" OR "avelumab" OR "ipilimumab" OR "tremelimumab" OR "relatlimab" OR "tiragolumab" OR "botensilimab" OR "camrelizumab" OR "sintilimab" OR "toripalimab" OR "tislelizumab" OR "penpulimab" OR "zimberelimab" OR "serplulimab" OR "sugemalimab" OR "envafolimab" OR "prolgolimab" OR "balstilimab" OR "retifanlimab" OR "cadonilimab" OR "ivonescimab" OR "CAR-T" OR "CAR T-cell*" OR "chimeric antigen receptor*" OR "CAR-NK*" OR "NK cell therap*" OR "TIL therapy" OR "tumor infiltrating lymphocyte*" OR "lifileucel" OR "TCR-T" OR "adoptive cell therap*" OR "oncolytic virus*" OR "oncolytic virotherap*" OR "T-VEC" OR "talimogene" OR "interleukin*" OR "cytokine therap*" OR "interferon*" OR "cancer vaccin*" OR "tumor vaccin*" OR "neoantigen vaccin*" OR "sipuleucel-T" OR "bispecific antibod*" OR "BiTE" OR "T-cell engager*" OR "tarlatamab" OR "amivantamab" OR "tebentafusp" OR "immune agonist*" OR "immune modulat*" OR "STING agonist*") AND ("cancer*" OR "oncolog*" OR "tumor*" OR "neoplasm*" OR "carcinoma*" OR "malignanc*" OR "sarcoma*" OR "melanoma*" OR "glioblastoma*" OR "neuroblastoma*" OR "mesothelioma*" OR "metastasis*" OR "metastatic") NOT ("leukemia*" OR "lymphoma*" OR "myeloma*" OR "hematologic*")

SUPPLEMENTARY MATERIAL

Supplementary Figure 1. Comparative subset analysis of bibliometric trends using the Scopus database.

(A) Annual publication output (2000–2025) derived from Scopus, demonstrating an exponential growth trajectory consistent with the primary WoSCC findings.

(B) The top 10 most productive countries/regions in the Scopus dataset, illustrating descriptive parallels with the leading contributions of China and the USA.

(C) The top 10 most productive journals in the field.

(D) VOSviewer keyword co-occurrence network map based on the top 20,000 most-cited documents in Scopus. Colors denote distinct thematic clusters: *Tumor Microenvironment & Biomarkers* (Cluster 1), *Inflammatory Mechanisms* (Cluster 2), and *Clinical Checkpoint Blockade* (Cluster 3).

(A)

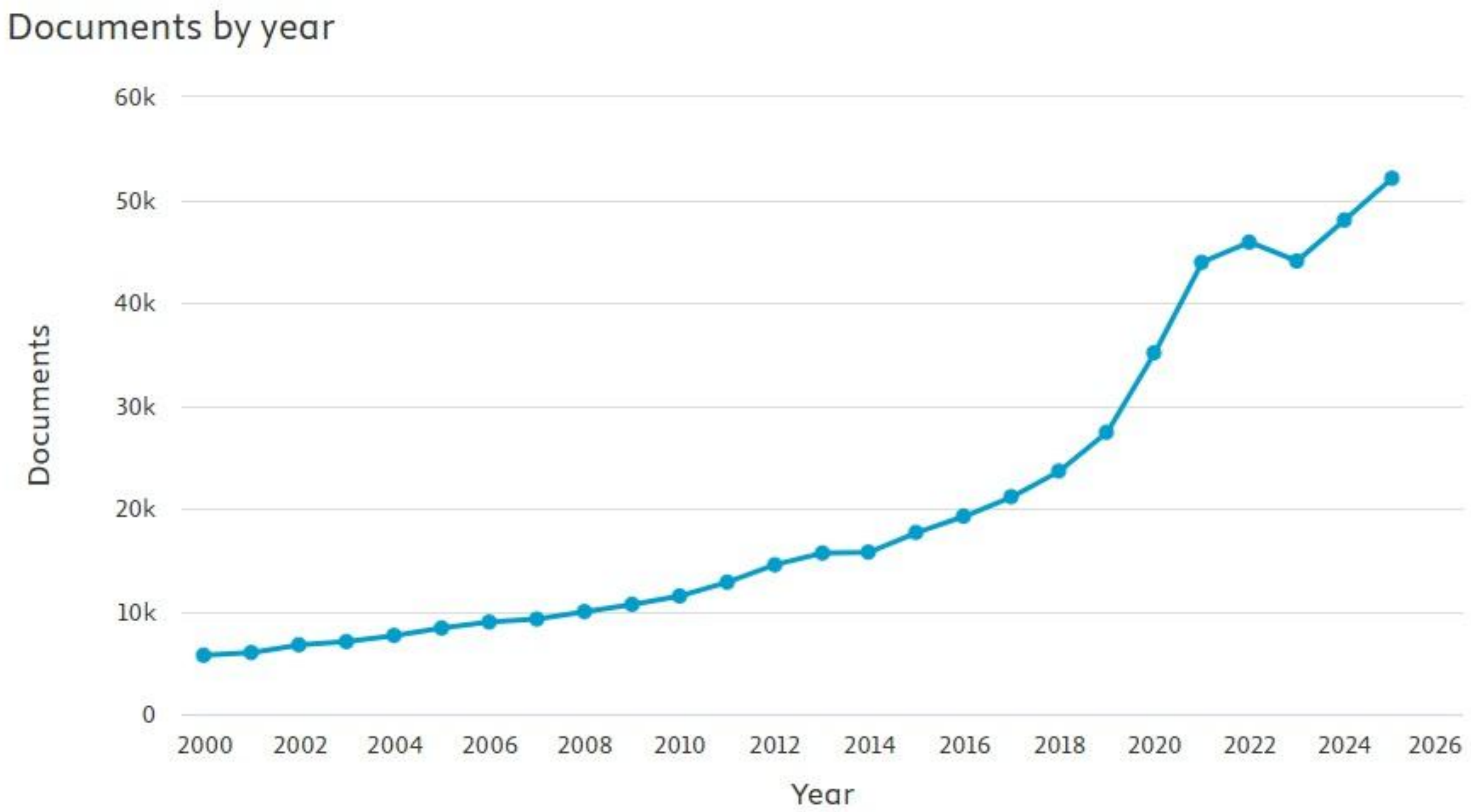

Documents by year
60k
50k
40k
30k
20k
10k
0
Documents
2000
2002
2004
2006
2008
2010
2012
2014
2016
2018
2020
2022
2024
2026
Year

(B)

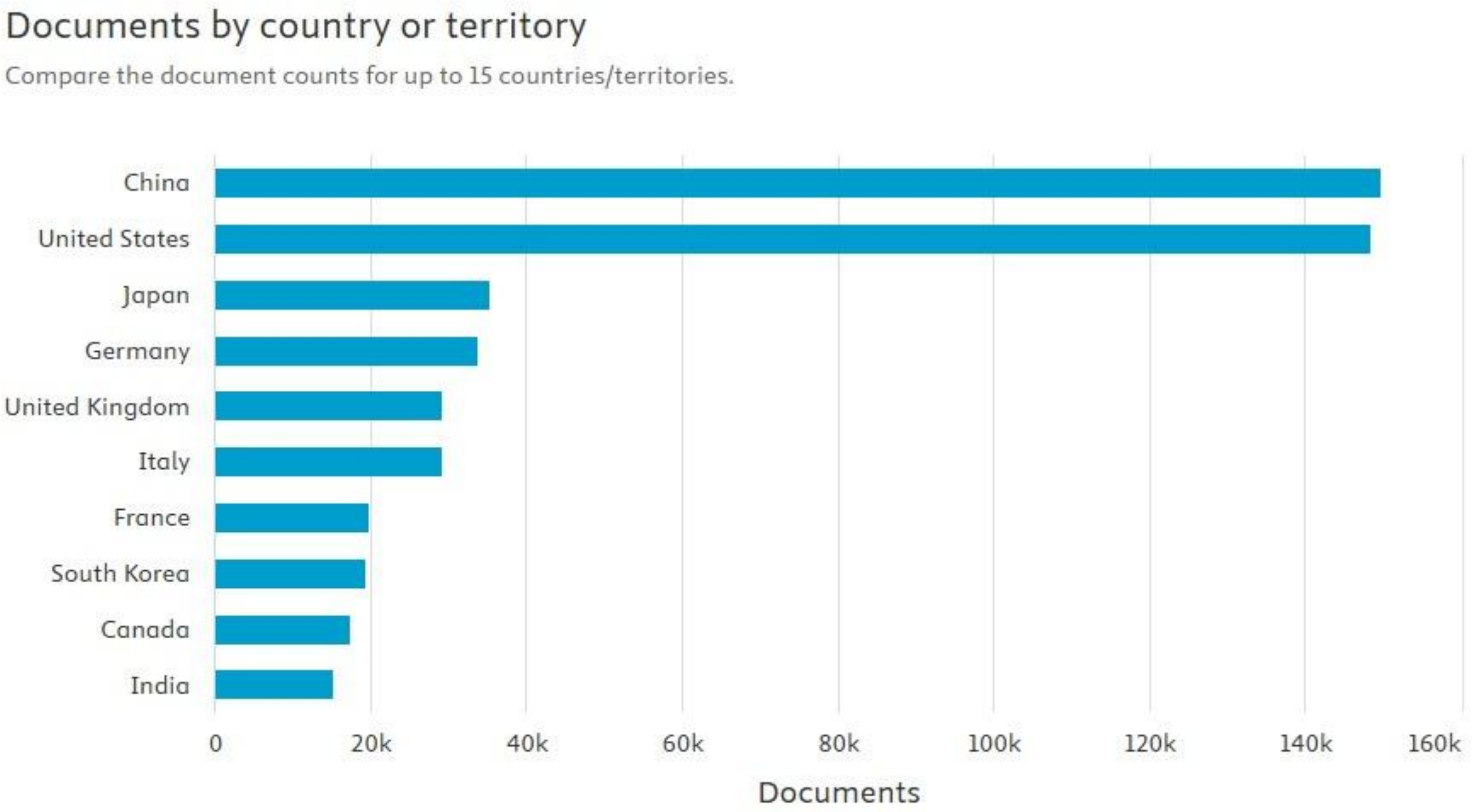

Documents by country or territory
Compare the document counts for up to 15 countries/territories.
China
United States
Japan
Germany
United Kingdom
Italy
France
South Korea
Canada
India
0
20k
40k
60k
80k
100k
120k
140k
160k
Documents

(C)

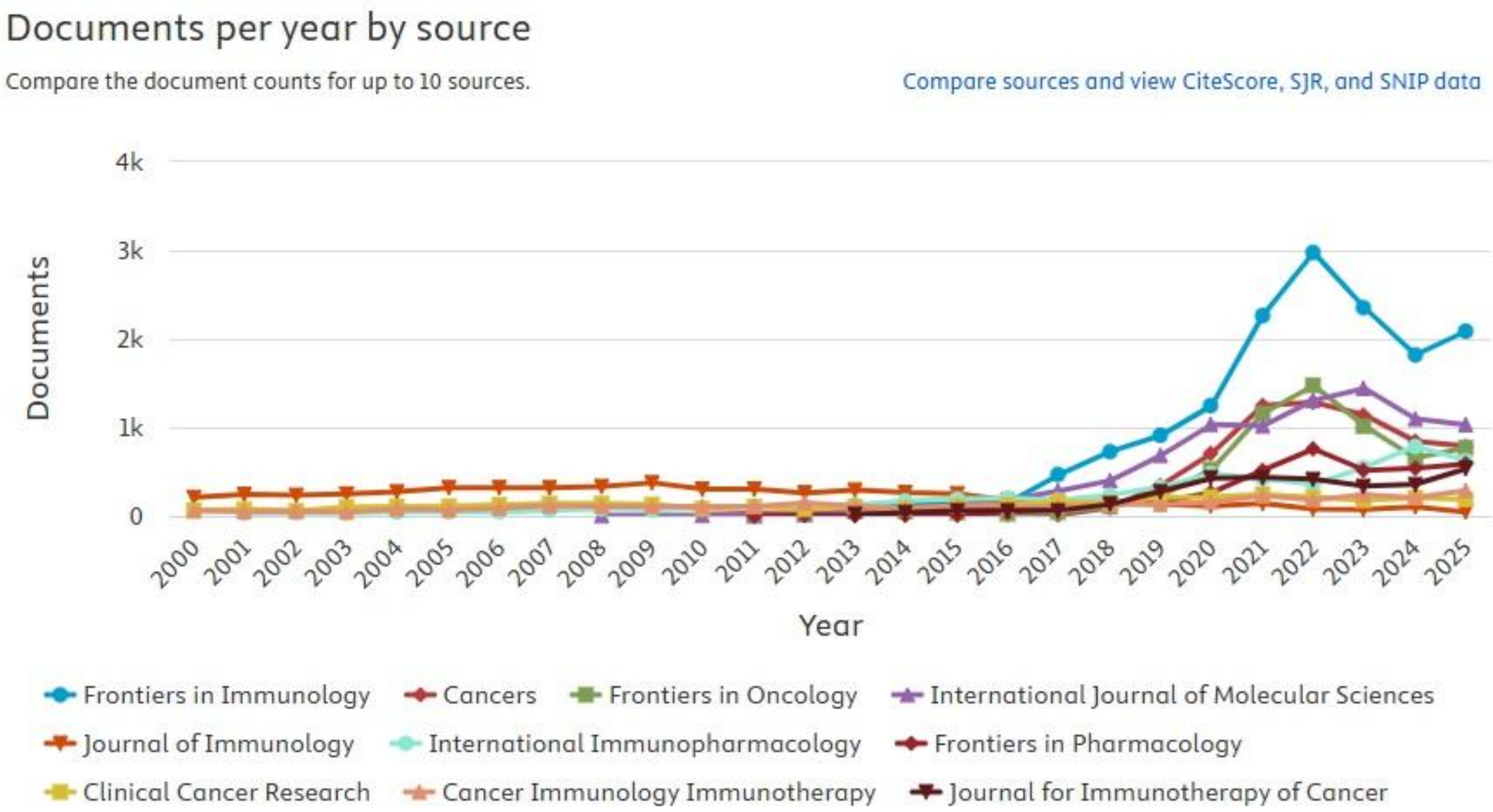

Documents per year by source
Compare the document counts for up to 10 sources.
Compare sources and view CiteScore, SJR, and SNIP data
4k
3k
2k
1k
0
Documents
2000
2001
2002
2003
2004
2005
2006
2007
2008
2009
2010
2011
2012
2013
2014
2015
2016
2017
2018
2019
2020
2021
2022
2023
2024
2025
Year
Frontiers in Immunology
Cancers
Frontiers in Oncology
International Journal of Molecular Sciences
Journal of Immunology
International Immunopharmacology
Frontiers in Pharmacology
Clinical Cancer Research
Cancer Immunology Immunotherapy
Journal for Immunotherapy of Cancer

(D)

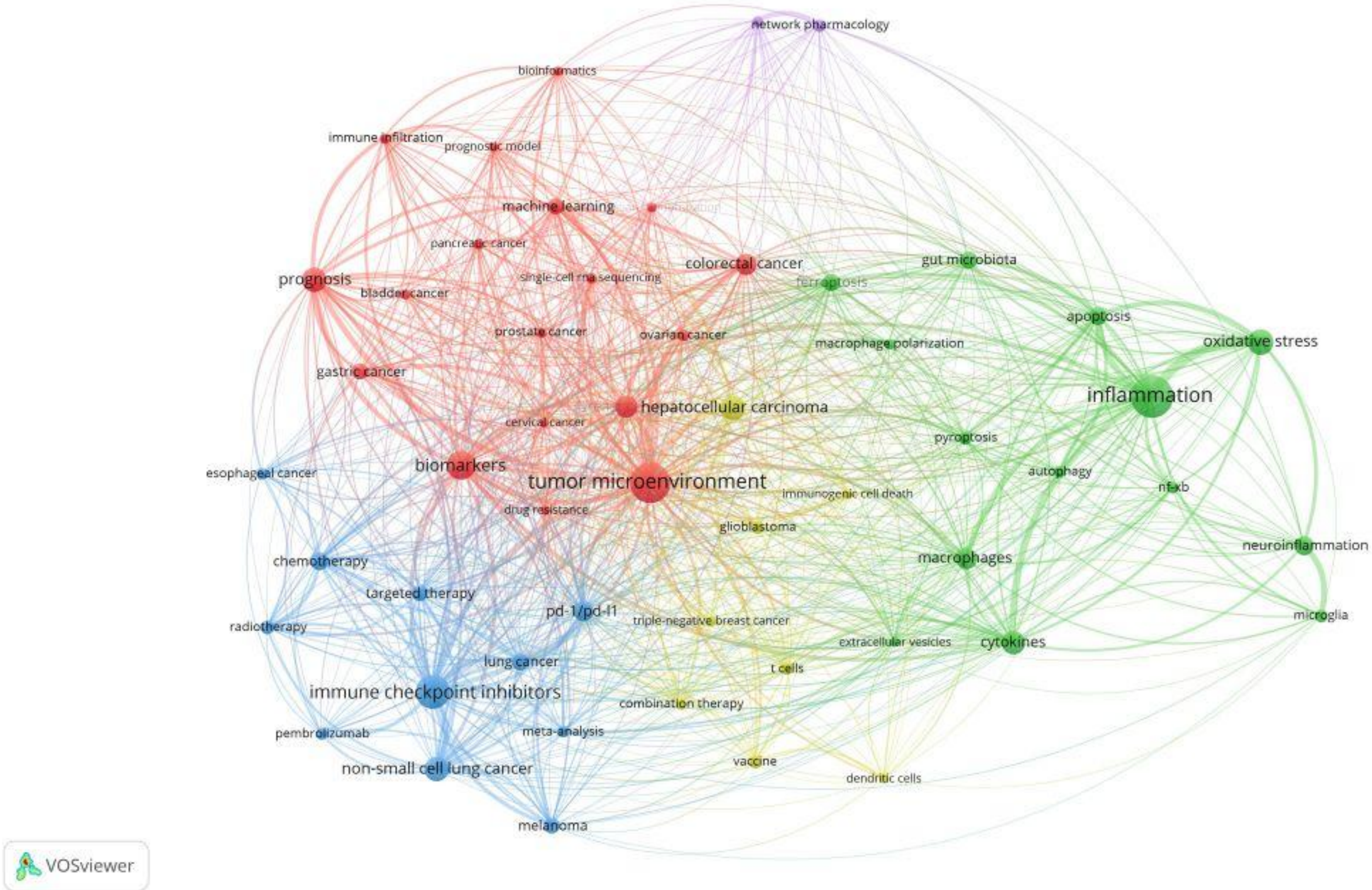

network pharmacology
bioinformatics
immune infiltration
prognostic model
machine learning
pancreatic cancer
prognosis
bladder cancer
single-cell rna sequencing
colorectal cancer
ferroptosis
gut microbiota
apoptosis
oxidative stress
prostate cancer
ovarian cancer
macrophage polarization
gastric cancer
inflammation
hepatocellular carcinoma
cervical cancer
pyroptosis
esophageal cancer
biomarkers
tumor microenvironment
autophagy
nf-kb
immunogenic cell death
drug resistance
glioblastoma
neuroinflammation
chemotherapy
macrophages
targeted therapy
pd-1/pd-l1
microglia
radiotherapy
triple-negative breast cancer
extracellular vesicles
cytokines
lung cancer
t cells
immune checkpoint inhibitors
combination therapy
pembrolizumab
meta-analysis
vaccine
non-small cell lung cancer
dendritic cells
melanoma
VOSviewer

Thesaurus

Replace by, Label

biomarkers, biomarker

vaccine, vaccines

vaccine, cancer vaccines

vaccine, cancer vaccine

vaccine, vaccination

tumor microenvironment, tumor microenvironment - tme

tumor microenvironment, tumor immune microenvironment

tumor microenvironment, ımmune microenvironment

tumor microenvironment, immune microenvironment

immune checkpoint inhibitors, immune checkpoint inhibitors (icis)

immune checkpoint inhibitors, immune checkpoint inhibitor

immune checkpoint inhibitors, ımmune checkpoint inhibitors

immune checkpoint inhibitors, ımmune checkpoint inhibitor

immune checkpoint inhibitors, immune checkpoint blockade

immune checkpoint inhibitors, checkpoint inhibitors

immune checkpoint inhibitors, checkpoint inhibitor

immune checkpoint inhibitors, pd-1 inhibitor

melanoma, metastatic melanoma

pd-1/pd-l1, pd-1

pd-1/pd-l1, pd-l1

immune checkpoint inhibitors, anti-pd-1

immune checkpoint inhibitors, anti-pd-l1

immune checkpoint inhibitors, anti pd-1

immune checkpoint inhibitors, anti pd-l1

non-small cell lung cancer, nsclc

non-small cell lung cancer, non-small-cell lung cancer

non-small cell lung cancer, non-small cell lung cancer (nsclc)

hepatocellular carcinoma, hcc

esophageal cancer, esophageal squamous cell carcinoma

il-6, interleukin-6

tnf-alpha, tumor necrosis factor alpha

tnf-alpha, tumor necrosis factor-alpha

il-18, interleukin-18

il-2, interleukin-2

il-12, interleukin-12

il-12, interleukin 12

il-10, interleukin-10

il-10, interleukin 10

il-8, interleukin-8

ifn-gamma, interferon-gamma

ifn-gamma, interferon gamma

ifn-alpha, interferon-alpha

ifn-alpha, interferon alpha

chimeric antigen receptor, chimeric antigen receptor - car

t cells, regulatory t cells

t cells, t cell

cytokines, cytokine

ipilimumab, ıpilimumab

dendritic cells, dendritic cell

inflammation, ınflammation

immune infiltration, ımmune infiltration

immunogenic cell death, ımmunogenic cell death

macrophages, macrophage
